# Molecular and Structural Characterization of Foam Proteins from *Mahanarva spectabilis* (Distant, 1909) (Hemiptera: Cercopidae) Nymphs Reveals Adaptive Features and Potential Targets for Pest Control

**DOI:** 10.1002/arch.70130

**Published:** 2026-02-17

**Authors:** Angelo José Rinaldi, Monique da Silva Bonjour, Ian de Paula Alves Pinto, Gabriely Teixeira Bhering Faria, Lucas Leal Lima, Marcela Chellini Pereira, Alexander Machado Auad, Jorge Fernando Pereira, Maria Goreti Almeida Oliveira, Humberto Josué de Oliveira Ramos

**Affiliations:** ^1^ Laboratory of Enzymology and Biochemistry of Proteins and Peptides, Department of Biochemistry and Molecular Biology Universidade Federal de Viçosa, UFV, BIOAGRO/INCT‐IPP Viçosa‐MG Brazil; ^2^ Embrapa Dairy Cattle Juiz de Fora Brazil; ^3^ Núcleo de Análise de Biomoléculas, NuBioMol Universidade Federal de Viçosa Viçosa‐MG Brazil

**Keywords:** AlphaFold structural modeling, biocontrol targets, insect extracellular matrix, plant defense

## Abstract

During its nymphal development, the spittlebug *Mahanarva spectabilis* (Distant, 1909) (Hemiptera: Cercopidae) secretes a persistent extracellular foam that functions as a multifunctional barrier against environmental stressors. In this study, we present a molecular and structural characterization of the foam proteins using LC‐MS/MS and AlphaFold‐based structural modeling. Although no significant differences were observed in total protein concentration across different host plant genotypes, proteomic analyses revealed the down‐regulation of specific high‐abundance proteins in nymphs feeding on resistant/moderately resistant grasses. This suggests a potential impairment of foam functionality and reduced nymphal fitness under field conditions. Peptides from individual SDS‐PAGE bands mapped to multiple distinct unigenes, indicating that proteins encoded by different transcripts share highly conserved sequence motifs, domain architectures, and structural folds. This was particularly evident for the most abundant protein, likely reflecting post‐translational modifications such as signal peptide cleavage, proteolytic processing, or alternative splicing. AlphaFold structural predictions revealed the presence of adhesive and matrix‐related domains, such as WSC, S‐layer, ankyrin repeats, and apolipophorin folds, across several foam proteins. The dominance of extended α‐helices and the predicted dimerization interfaces reinforce the hypothesis that these proteins participate in the formation of supramolecular scaffolds essential for the mechanical stability and adhesion of the foam. Collectively, these findings suggest that *M. spectabilis* foam proteins have undergone evolutionary specialization to assemble a multifunctional extracellular matrix that ensures nymphal protection. These insights highlight potential molecular targets for novel pest control strategies and contribute to the broader understanding of insect‐derived extracellular secretions with biomimetic relevance.

## Summary

- Foam proteins of *Mahanarva spectabilis* nymphs form a conserved extracellular matrix with adhesive and structural domains.
- Key high‐abundance proteins are down‐regulated in resistant/moderately resistant host plants, potentially impairing foam stability.
- These proteins represent promising molecular targets for innovative pest control strategies.

## Graphical abstract

Nymphs of *Mahanarva spectabilis* produce a protective foam that supports survival, reduces plant damage, and contributes to adult population establishment. Proteomic profiling combined with protein sequencing and structural modeling revealed foam proteins enriched in functional domains associated with environmental resilience. Key features include extended α‐helices and dimerization promoting supramolecular cohesion, adhesive and matrix‐forming motifs linked to stress protection, conserved structural motifs, and bacterial‐like/toxin signatures suggesting roles in microbial community regulation. Additional oxidoreductases indicate active reactive‐oxygen control. Together, these molecular traits highlight the foam as a multifunctional biochemical barrier that integrates mechanical stability, microbial modulation, and environmental protection.

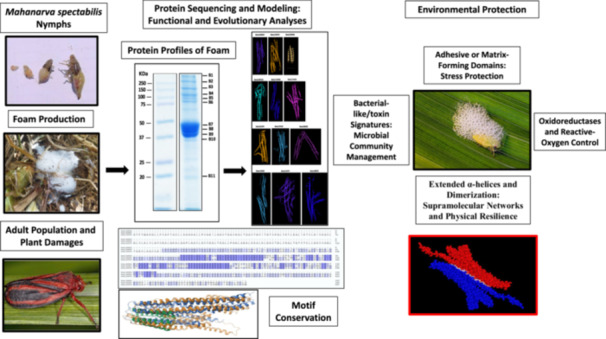

## 1 Introduction

Forage grasses are essential components of tropical livestock systems, serving as the primary source of nutrition for ruminants. In Brazil, species such as *Brachiaria* and *Cenchrus purpureus (syn. Pennisetum purpureum)* are commonly used as pastures due to their high biomass productivity and adaptability to diverse soil and climatic conditions (Euclides et al. 2014; Schneider et al. 2016). However, their productivity and persistence are severely compromised by infestations of insect pests, particularly the spittlebug *Mahanarva spectabilis* (Hemiptera: Cercopidae). This pest causes substantial economic losses to Brazil's livestock industry, with millions of dollars in annual damages due to reduced pasture yields and increased management costs (Oliveira et al. 2014; Hollmann and Peck 2002; Dias et al. 2019). Both nymphs and adults of this species cause damage to grasses. Adults feed on leaf sheaths and culms, injecting phytotoxic saliva that induces vascular dysfunction and suppresses photosynthetic efficiency (Silva 2012; Resende 2012; Pabón et al. 2007). Meanwhile, nymphs feed on the roots and rhizomes while remaining hidden inside a foam mass, resulting in weakened plants, biomass loss, and pasture degradation (Valério and Nakano 1988; Tonelli 2019).

The foam secreted by *M. spectabilis* nymphs plays a vital role in their development. This aerated matrix provides physical protection from predators, thermal buffering, and moisture retention, thereby creating a stable microenvironment that supports feeding and growth (Tonelli 2019; Chen et al. 2018; Capinera 2008). Compositional analyses of the foam indicate that it is composed primarily of water (approximately 90–95%), with the remaining fraction consisting of lipids, carbohydrates, proteins, and surfactants (Sahayaraj et al. 2021; Tonelli 2019). These components are believed to contribute not only to the foam's structural stability and water retention capacity but also to its biological properties, such as antimicrobial activity and enzymatic regulation. Recent studies suggest that the foam may also modulate plant defense responses, potentially influencing the insect's ability to colonize host plants (Barros et al. 2021; Sahayaraj et al. 2021).

Despite its ecological and physiological relevance, the biochemical composition of *M. spectabilis* foam remains poorly characterized as previous studies have mostly focused on morphological and biological aspects (Tonelli et al. 2019; Chen et al. 2018). Studies highlight that certain grass cultivars, such as *B. brizantha* and *P. purpureum*, exhibit antixenosis and antibiosis mechanisms that affect insect feeding and development, potentially altering the quantity or composition of the foam produced (Barros 2018; Parsa et al. 2011; Lapointe et al. 1992). For instance, resistant genotypes may reduce the expression of key foam proteins, impacting nymphal fitness and offering insights into pest control strategies (Espitia Buitrago et al. 2022; Niere et al. 2001; José Rinaldi et al. 2026).

In other Cercopidae species, few reports have explored the protein content of foam secretions. For example, studies in *Philaenus spumarius* and *Aeneolamia varia* have identified components such as mucopolysaccharides, surfactant proteins, and enzymes related to microbial defense (Cameirão et al. 2025; Espitia Buitrago et al. 2022; Kiragu et al. 2014; Del Campo et al. 2011). Comparative analyses with *M. spectabilis* could reveal conserved or unique protein profiles, enhancing our understanding of foam evolution and function across spittlebug species. However, proteomic analyses remain rare and fragmented, limiting our understanding of how foam constituents may vary among species or relate to host plant use.

We hypothesize that the foam of *M. spectabilis* not only serves as a physical barrier but also reflects physiological adaptations to host plant interactions. Specifically, the foam proteome may differ depending on whether nymphs develop on resistant or susceptible grass genotypes. These differences could contribute to the insect's survival, modulate the host's defense response, or reflect a biochemical feedback loop in resistant interactions. Identifying these proteins could enable targeted pest control strategies, such as disrupting foam stability or enhancing plant resistance mechanisms.

To investigate this hypothesis, we conducted a proteomic analysis of foam secretions produced by *M. spectabi*lis nymphs reared on four forage grass genotypes, one resistant, one moderately resistant and two susceptible. We employed shotgun proteomics coupled with Orbitrap‐based LC‐MS/MS, transcriptomic analysis by RNAseq and applied structural modeling and functional annotation tools, including AlphaFold3, to characterize the identified proteins. By proteomic and structural biology techniques, including AlphaFold3 for high‐accuracy protein structure prediction, this study advances our understanding of insect‐plant interactions and their implications for sustainable pest management. Our findings provide insights into the characterization and functional composition of spittlebug foam and its potential roles in insect–plant interactions. Furthermore, this knowledge could inform integrated pest management strategies which may improve the efficacy in controlling *M. spectabilis* populations in pasture and sugarcane systems (Mascarin et al. 2019; Iwanicki et al. 2019). By elucidating how foam proteins interact with such agents, we can optimize their application for environmentally friendly pest control.

## 2 Materials and Methods

### 2.1 Assays of Plant Infestation and Foam Sampling

Foam samples produced by fourth and fifth instar nymphs of *Mahanarva spectabilis* were provided by Embrapa Dairy Cattle (Juiz de Fora, MG, Brazil). The grasses were propagated in 1 L pots containing a soil mixture and commercially available Plantmax substrate. The 4th and 5th instar nymphs were kept for 24 h on 3 to 4‐month‐old plants covered with organza bags to prevent their escape, maintained in a glasshouse at 25°C and 70% relative humidity until foam formation. A total of twelve samples were collected (one foam sample represent the foam from three infested plants) from nymphs infesting four forage genotypes: *Urochloa brizantha (syn. Brachiaria brizantha)* (BRI), *Urochloa decumbens (syn. Brachiaria decumbens)* (DEC), and *Cenchrus purpureus (syn. Pennisetum purpureum)* cultivars (Pioneiro—PIO and Roxo Botucatu—ROXO), with three biological replicates per genotype. The foam samples were kept on ice and then frozen in liquid nitrogen and stored at −80°C

The samples were transferred to the Center for Biomolecules Analysis at Federal University of Viçosa (Viçosa, MG, Brazil). The foam samples were centrifuged at 10,000 × g for 10 min and transferred to new 50 mL Falcon tubes. The resulting supernatants were then transferred to 1.5 mL microtubes. Each sample volume was standardized to 500 µL using Milli‐Q water, and the samples were dried in a speed vacuum concentrator.

### 2.2 Protein Extraction and Quantification

Proteins were solubilized in 100 µL of 10% sodium dodecyl sulfate (SDS) buffer. After homogenization, the samples were centrifuged at 12,000 × g for 15 min at 4°C, and the supernatants were collected. Protein concentration was determined using the bicinchoninic acid (BCA) assay, with bovine serum albumin (BSA) as the standard, measured at 562 nm using a microplate reader spectrophotometer.

### 2.3 Protein Separation by SDS‐PAGE and Densitometric Analysis

Protein samples extracted from the foam secreted by *Mahanarva spectabilis* nymphs were separated by one‐dimensional sodium dodecyl sulfate‐polyacrylamide gel electrophoresis (1D SDS‐PAGE) using a 12% resolving gel under denaturing conditions. Equal amounts of total protein were loaded into each well, and electrophoresis was performed at constant voltage until optimal separation was achieved. Following electrophoresis, gels were stained with Coomassie Brilliant Blue R‐250 and subsequently destained to visualize protein bands.

Densitometric analysis of individual protein bands was carried out using the ImageMaster 2D Platinum software (GE Healthcare), which enables quantification based on integrated optical density (IOD). The intensity of each band was determined by summing the pixel intensity values within the defined band area, after automated background subtraction. The relative abundance of each band was calculated as a proportion of the total IOD detected in the respective lane. The resulting values were used to estimate the comparative abundance of proteins across biological replicates and treatments.

Statistical analysis was performed using one‐way analysis of variance (ANOVA) followed by post hoc multiple comparison tests to assess differences in protein band intensities among treatments. Significance was determined at *p* < 0.05, and results were used to evaluate the differential abundance of proteins across experimental groups.

### 2.4 Transcriptome Sequencing and Protein Database Construction

Total RNA was extracted from *Mahanarva spectabilis* nymphs using TRIzol® Reagent (Invitrogen), following an optimized protocol for insect samples (Bonjour et al. 2024). Briefly, tissue samples were homogenized in TRIzol, and RNA was isolated according to the manufacturer's instructions, including phase separation, RNA precipitation with isopropanol, washing with 75% ethanol, and resuspension in nuclease‐free water. The extracted RNA was then used to generate a transcriptomic database for protein identification.

RNA quality was assessed using NanoDrop, Qubit 2.0, and BioAnalyzer Agilent 2100. Only high‐quality RNA samples were processed for mRNA library construction. Polyadenylated mRNA was isolated using oligo(dT) magnetic beads, randomly fragmented, and reverse‐transcribed into cDNA using random hexamers. Second‐strand synthesis was performed, and the resulting cDNA was purified, end‐repaired, A‐tailed, and ligated to adapters. Fragments of 300–400 bp were selected and PCR‐amplified to generate cDNA libraries. Libraries were quantified with Qubit and Agilent Bioanalyzer, and sequenced using the Illumina platform.

Raw reads were processed to remove adaptors and low‐quality sequences, generating clean FASTQ files, deposited in the NCBI Sequence Read Archive (SRA) under BioProject PRJNA1404840. High‐quality reads were assembled de novo into contigs using optimized transcriptome assemblers. Assembled contigs were then functionally annotated by aligning predicted protein sequences against public databases (NR, Swiss‐Prot, COG, KOG, KEGG) using DIAMOND. Gene Ontology (GO) terms were assigned via InterProScan, and Pfam domains were identified using HMMER, facilitating the construction of a comprehensive reference protein database for downstream proteomic analysis.

### 2.5 Protein Identification by LC‐MS/MS

Individual protein bands of interest were excised manually from Coomassie‐stained SDS‐PAGE gels using sterile scalpels. Gel pieces were washed sequentially with 50 mM ammonium bicarbonate and acetonitrile (1:1) to remove residual stain. Samples were then reduced with 10 mM dithiothreitol (DTT) at 56°C for 30 min and alkylated with 55 mM iodoacetamide (IAA) in the dark at room temperature for 30 min. Following alkylation, gel fragments were dehydrated with acetonitrile, rehydrated in trypsin solution (12.5 ng/μL in 50 mM ammonium bicarbonate), and incubated overnight at 37°C for enzymatic digestion.

The dried peptide samples were reconstituted in 50 µL of 0.1% formic acid and analyzed using a nano‐UHPLC system (UltiMate R 3000, Dionex, San Jose, USA) equipped with an Acclaim PepMap100 C18 Nano‐Trap column (100 µm i.d. × 20 mm, 5 µm, 100 Å; Thermo Scientific, Waltham, MA, USA) and an analytical Acclaim PepMap100 C18 RSLC column (75 µm i.d. × 150 mm, 2 µm, 100 Å; Thermo Scientific, Waltham, MA, USA), in tandem with the trap column, operating at a constant flow rate of 0.3 µL min⁻¹. The solvents used were: (A) 0.1% formic acid (HPLC grade, JTBaker, Mexico) and (B) 80% acetonitrile with 0.1% formic acid (HPLC grade, JTBaker, Mexico). A multistep gradient was applied as follows: an initial conditioning step with 3.8% B for 3 min, followed by a linear increase from 3.8% to 30% B over 120 min, then from 30% to 55% B by 150 min. A final ramp to 99% B was completed at 162 min, followed by reconditioning with 3.8% B until 180 min.

Spectral data were acquired using a Q‐Exactive mass spectrometer (Thermo Scientific, Bremen, Germany) operating in full‐scan/MS2 mode. The nanospray flex ion source (Thermo Scientific) was set to 3.8 kV in positive mode, with a capillary temperature of 250°C and S‐ lens RF level set to 55.

The Data‐Dependent Acquisition (DDA) method was configured to select the top 12 ions with charge states between +2 and +4 within a 1.2 m/z isolation window. Survey scans were acquired at a resolution of 70,000 with a mass range of 300–1800 m/z.

Protein identification was carried out using PEAKS Studio software (Bioinformatics Solutions Inc.), combining de novo peptide sequencing with database‐dependent searching. The custom database used for peptide matching was derived from a *Mahanarva spectabilis* transcriptome assembly generated by RNA‐Seq (Illumina). Search parameters included a precursor mass tolerance of 10 ppm and fragment mass tolerance of 0.02 Da. Protein identifications were accepted only if they met a false discovery rate (FDR) threshold of 0.1% at the peptide level and 1% at the protein level, and were supported by at least three unique peptides.

### 2.6 Sequence Alignment and Phylogenetic Analysis

To assess the sequence similarity among proteins identified in the foam of *M. spectabilis*, multiple sequence alignment was performed using the UniProt alignment tool (https://www.uniprot.org/align). The alignment was conducted using the Clustal Omega algorithm, which applies a seeded guide tree and HMM profile‐profile techniques for accurate multiple sequence alignment. The method supports high‐throughput comparisons and is optimized for both speed and alignment quality. The resulting alignments were used to generate phylogenetic trees and visualize the clustering relationships among protein sequences.

### 2.7 AlphaFold3 Modeling and Structure Prediction

The structural modeling and functional predictions of foam‐derived proteins were performed using the AlphaFold3 implementation hosted on the 310. AI platform (https://310.ai/copilot/ad577d51-748e-4657-919f-478b79ae231a). For each identified protein sequence, the AlphaFold3 pipeline generated high‐confidence structural models based on deep learning approaches that incorporate physical and evolutionary constraints. The platform provided per‐residue confidence scores, predicted aligned error (PAE), and TM‐scores, which were used to assess the quality of the generated structures. Protein models with TM‐scores > 0.5 were considered to have reliable global folds. Structural analogs were identified by comparison to experimentally resolved structures in the PDB, allowing inference of potential functions through structural homology.

## 3 Results

### 3.1 Total Protein Concentration in Foam Samples

Total protein concentration in the foam produced by *Mahanarva spectabilis* nymphs was quantified from samples collected on different forage grass genotypes, including BRI, PIO, ROXO, and DEC. The average protein concentrations ranged from ~0.49 to 0.66 µg/µL across all genotypes (Table 1). Although some variation was observed between replicates, statistical analysis revealed no significant differences in total protein concentration among the different plant genotypes (*p* > 0.05). These findings suggest that the host plant genotype does not substantially influence the overall protein content of the foam secreted by *M. spectabilis* nymphs.

**Table 1:** Total protein concentration of the foam produced by of *Mahanarva spectabilis* nymph infesting different forage grass genotypes.

Genotype	Replicates	Concentration (µg/µL)^a^
BRI	BRI 1	0.5825 ± 0.003
	BRI 2	0.5718 ± 0.002
	BRI 3	0.5781 ± 0.004
PIO	PIO 1	0.6059 ± 0.005
	PIO 2	0.6625 ± 0.006
	PIO 3	0.6183 ± 0.004
ROXO	ROXO 1	0.5343 ± 0.003
	ROXO 2	0.4919 ± 0.002
	ROXO 3	0.5403 ± 0.005
DEC	DEC 1	0.6657 ± 0.007
	DEC 2	0.5140 ± 0.003
	DEC 3	0.5442 ± 0.004

^a^ No significative diferences was observed.

### 3.2 Differential Abundance of Protein SDS‐PAGE Profiles Across Host Grass Genotypes

**Figure 1:**
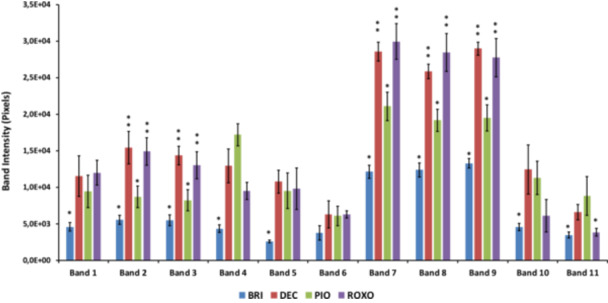
Relative abundance of 11 protein bands identified in foam secretions of *Mahanarva spectabilis* nymphs collected from four forage grass genotypes: BRI (resistant), PIO (moderately resistant), and ROXO and DEC (susceptible). Band intensities were determined by densitometric analysis of SDS‐PAGE gels. Bars represent mean values ± standard error of the mean (SEM). Bars without asterisks indicate no statistically significant differences. Bars marked with one asterisk (*) are significantly different from those marked with two asterisks (**), based on post hoc mean comparison tests (*p* < 0.05).

Densitometric analysis of SDS‐PAGE gels revealed differential abundance patterns for 11 protein bands in foam secretions from *Mahanarva spectabilis* nymphs feeding on different forage grass genotypes (Figure 1 and Figure S1). Among the genotypes analyzed, BRI is considered resistant to insect attack, PIO is moderately resistant while ROXO and DEC are susceptible.

Significant differences in band intensities were observed between resistant/moderately resistant and susceptible genotypes. Specifically, bands 2, 3, 7, 8 and 9 exhibited lower relative abundance in resistant/moderately resistant genotypes (BRI and PIO), suggesting a possible correlation between these protein components and plant‐mediated resistance responses. Conversely, bands 1, 5, 10, and 11 showed a tendency to increased abundance in the foam of nymphs associated with susceptible genotypes (DEC and ROXO), potentially reflecting physiological or immunological differences in response to more permissive hosts. These differences in protein abundance may impair the foam's protective function on resistant/moderately resistant plants, potentially reducing nymph survival and offering insights for developing targeted pest control strategies, such as disrupting foam stability.

These results support the hypothesis that host plant genotype can influence the composition of foam‐associated proteins, potentially reflecting adaptive responses of *M. spectabilis* to resistant/moderately resistant versus susceptible genotypes.

### 3.3 Protein Identification by LC‐MS/MS From Differential Bands

**Figure 2:**
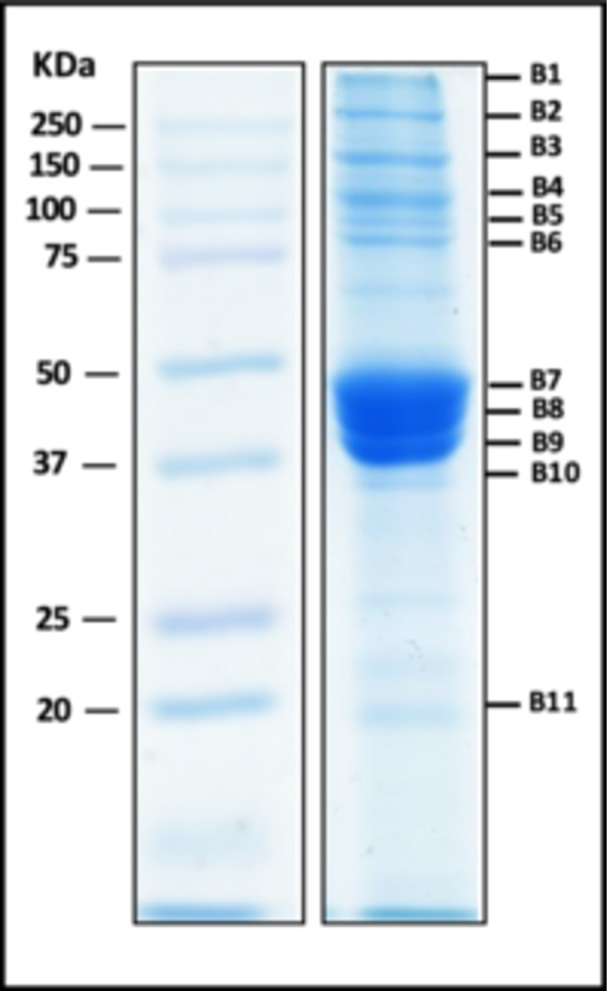
SDS‐PAGE gel image of the protein bands showing higher relative abundance in the foam of *Mahanarva spectabilis* nymphs. The sample corresponds to foam collected from nymphs reared on the PIO genotype (moderately resistant host). This gel serves as a representative visualization of total foam protein complexity and was used for illustrative purposes. Comparative proteomic analyses across all four genotypes (PIO, ROXO, BRI, DEC) were conducted via LC‐MS/MS, which offers greater resolution and sensitivity for quantitative comparisons.

The 11 protein bands with differential abundance across forage grass genotypes (Figure 1), visualized in Figure 2 and corresponding to distinct molecular weights on SDS‐PAGE, were excised and subjected to in‐gel tryptic digestion followed by LC‐MS/MS analysis. Protein identification was performed using PEAKS Studio against a transcriptome‐derived *Mahanarva spectabilis* protein database. The use of a transcriptome‐derived database is critical for non‐ model organisms like *M. spectabilis*, which lack a fully sequenced and annotated genome, enabling protein identification based on expressed gene sequences.

While SDS‐PAGE provides a visual overview of foam protein composition, its limited resolution restricts its use in comparative studies. Therefore, a representative gel from the PIO genotype was selected for illustrative purposes (Figure 2), given its performance as a moderately resistant host. For genotype‐level comparisons, high‐resolution quantitative proteomics via LC‐MS/MS was employed to assess differential protein abundance with greater specificity and sensitivity. Accordingly, SDS‐PAGE gels for all four genotypes were not generated or presented, as LC‐MS/MS provided superior resolution for comparative proteomic analysis.

In several bands, more than one protein was identified by PEAKS, reflecting the co‐ migration of multiple proteins with similar molecular weights. In Table 2, we present for each band the protein with the highest number of unique peptides and/or the greatest sequence coverage, parameters typically indicative of the most abundant protein in the sample. Additional proteins identified with lower coverage or peptide counts are provided in the supplementary materials. These results provide insight into the predominant protein constituents of each foam‐ associated band and serve as the basis for functional and structural annotation.

**Table 2:** Analysis of proteins of foam produced by *M. Spectabilis* nymph identified from 1D‐SDS‐PAGE 1DE‐gels by LC/MS.

Protein band	MW KDa—observed^a^	Gene^b^	MW Da – CDS^c^
B1	400	Gene|115205	190720
B2	270	Gene|37310	182521
B3	150	Gene|52303	145860
B4	100	Gene|105156	106270
B5	90	Gene|14174	88435
B6	80	Gene|49576	87976
B7	49	Gene|122372	50572
B8	45	Gene|122372	50572
B9	40	Gene|122372	50572
B10	34	Gene|105027	34923
B11	16	Gene|105031	14445

^a^ Values estimated from SDS‐PAGE gels.

^b^ Proteins identified by PEAKS Studio, showing higher coverage and number of identified peptides.

^c^ Values obtained from coding sequences (CDSs) generated through RNA‐Seq analysis.

When comparing the observed molecular weight from SDS‐PAGE with the theoretical molecular weight predicted from coding sequences (CDSs), several discrepancies were noted. In many cases, the observed molecular weight was lower than expected, suggesting that these proteins may undergo post‐translational modifications such as signal peptide cleavage, proteolytic processing, or truncation (Table 2). For example, Gene|122372 was identified in three bands (B7–B9), each with progressively decreasing molecular weights (~49, ~45, and ~40 kDa), despite encoding a full‐length protein of 50.57 kDa. This strongly supports the occurrence of protein isoforms or processing events that generate structurally related variants.

Moreover, the recurrence of certain genes across multiple bands, such as Gene|122372 and Gene|105027, highlights the presence of abundant, multifunctional proteins in the foam proteome. These may represent key structural components involved in foam stabilization, dimerization, or interaction with host or microbial molecules. Such redundancy or overlap also reinforces the hypothesis of functional convergence among foam proteins, even when derived from distinct transcriptomic sources.

Functional annotation of the identified proteins was attempted using multiple databases and tools, including Gene Ontology (GO), KEGG Orthology (KO), KOG, and Pfam. However, no functional matches were retrieved, likely due to the lack of homologous sequences in available reference protein databases. As a result, all highly abundant identified proteins were categorized as ‘unknown function’. This limitation highlights the need for expanded genomic and proteomic databases for hemipteran insects. Future studies could employ advanced structural modeling, such as AlphaFold3, to predict protein functions based on conserved domains or motifs and conduct functional assays to elucidate their roles in foam formation, stability, or interaction with host plants.

### 3.4 Phylogenetic and Sequence Similarity Analysis

**Figure 3:**
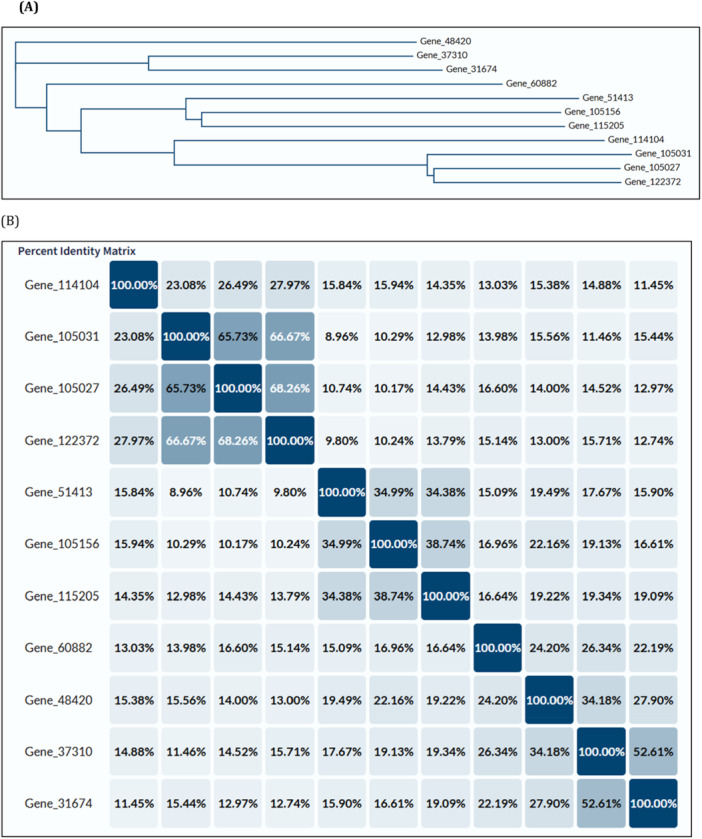
(A) Phylogenetic tree and (B) pairwise sequence identity matrix of the most abundant proteins identified in foam secretions of *Mahanarva spectabilis* nymphs. Proteins were aligned using the Clustal Omega algorithm via the UniProt alignment tool (https://www.uniprot.org/align).

Matrix‐based analyses such as sequence identity and phylogenetic clustering offer critical insights into the evolutionary and structural organization of foam proteins. The identity matrix (Figure 3B) quantifies pairwise similarity between protein sequences, with higher percentages indicating greater sequence conservation. These results suggest that many of the most abundant proteins share conserved motifs or domains, supporting potential functional redundancy or complementarity. In Figure 3A, the phylogenetic tree generated via Clustal Omega shows three distinct clusters of proteins with elevated intra‐group similarity. These clusters likely represent gene families or paralogous expansions, which may have evolved to fulfill specific structural or protective roles within the foam.

**Figure 4:**
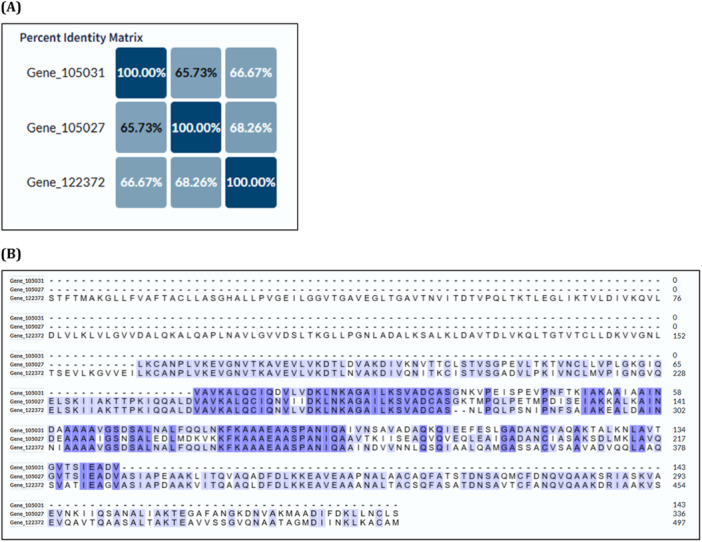
(A) Multiple sequence alignment of the three most abundant proteins in Cluster 01 as identified by LC‐ MS/MS. (B) Heatmap showing pairwise amino acid identity among these proteins. Sequence alignment was performed using the UniProt alignment tool (Clustal Omega algorithm).

**Figure 5:**
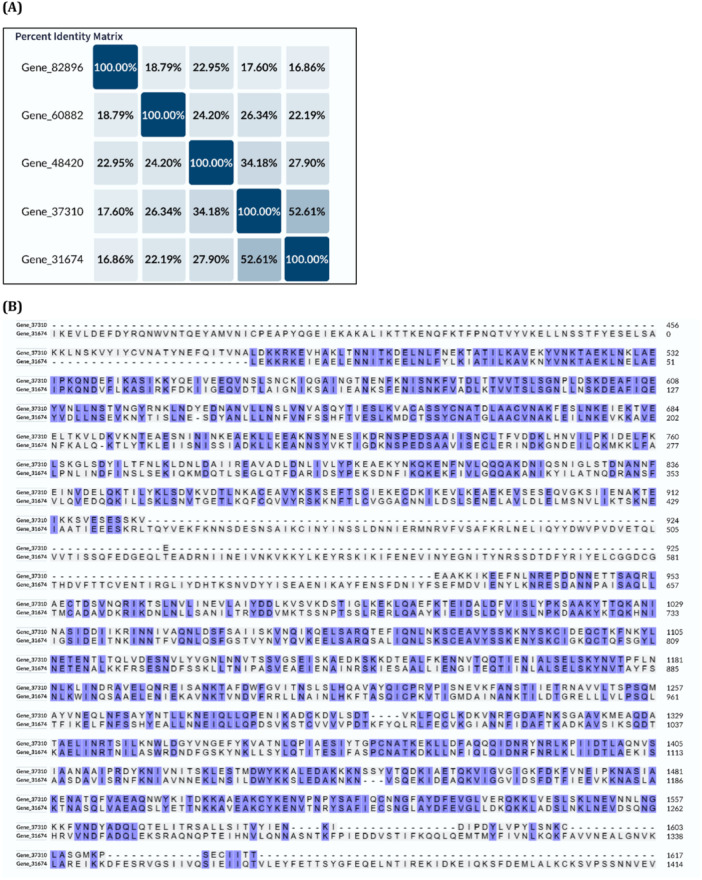
(A) Multiple sequence alignment of the three most abundant proteins in Cluster 02 as identified by LC‐ MS/MS. (B) Heatmap showing pairwise amino acid identity among these proteins. Sequence alignment was performed using the UniProt alignment tool (Clustal Omega algorithm). Only regions showing similarity are indicated.

Importantly, this grouping is supported by multiple sequence alignments (Figures 4A, 5A **)**, which reveal conserved residues and domain architecture despite differences in protein length or migration on SDS‐PAGE. The consistent detection of conserved domains in Clusters 01 and 02 indicates selective evolutionary pressure to maintain functional elements essential for foam‐mediated protection. The presence of isoforms, such as the ones derived from Gene|122372 in bands B7, B8, and B9, further suggests complex regulation through post‐ translational modification or alternative splicing, a hypothesis reinforced by signal peptide predictions (Figure S3).

The most abundant proteins identified in foam samples were subjected to multiple sequence alignment and phylogenetic analysis using the UniProt alignment tool (https://www.uniprot.org/align). The Clustal Omega algorithm was employed to generate a guide tree and compute pairwise sequence similarities. Clustal Omega is a widely used tool for aligning multiple protein sequences, enabling the identification of evolutionary relationships and conserved regions. The resulting phylogenetic tree (Figure 3A) illustrates the evolutionary relationships among the proteins, while Figure 3B presents the percentage sequence identity matrix.

Three distinct clusters were observed in the phylogenetic tree, each comprising proteins with higher intra‐group sequence similarity, composed of following proteins: Gene|105027, Gene|122372, Gene|105031, Gene|114174; Gene|105156, Gene|115205, Gene| 51413; Gene|31674, Gene|37310, Gene|48420. This clustering suggests possible shared ancestry or conserved functional features among specific subsets of foam proteins. These clusters may reflect gene family expansions or functional convergence, potentially linked to the foam's role in protecting nymphs from environmental stresses or facilitating host plant interactions. The presence of these similarity‐based groups may result from functional convergence, structural conservation, or gene family expansion events that are likely relevant to the biology of *M. spectabilis* nymphal foam secretion. Understanding these relationships could guide the identification of key proteins for targeted pest control strategies, such as disrupting foam stability.

### 3.5 Conserved Features Among Clustered Proteins

Detailed sequence comparison among the three proteins grouped in Cluster 01 (Figure 4A) revealed a high degree of similarity, particularly in specific conserved regions (Figure 4B). Despite differences in the overall length of the polypeptide chains, several conserved domains were identified across all three sequences, indicating the potential retention of shared structural or functional features. These conserved domains likely correspond to motifs critical for foam stability, adhesion, or interaction with environmental factors, reflecting evolutionary pressure to maintain essential functions.

This high similarity among discrete sequence regions supports the hypothesis of functional conservation, possibly reflecting evolutionary pressure to maintain roles essential for foam function or insect physiology. Such conservation suggests that these proteins could be targeted to disrupt the foam's protective properties, offering potential for pest management strategies.

Additionally, the detection of isoforms derived from the same unigene, such as Gene|122372, supports the occurrence of post‐translational modifications or alternative splicing, mechanisms that can generate structurally related proteins with distinct biophysical properties or regulatory functions. These findings suggest that even in the presence of structural divergence or varied molecular weights, key functional domains may be preserved, contributing to the observed similarities in the foam proteome of *M. spectabilis*.

The phylogenetic tree illustrates the clustering of proteins into three distinct groups with higher intra‐group sequence similarity. Percent identity values in the matrix reflect the degree of similarity between aligned protein pairs, supporting the existence of conserved structural or evolutionary relationships.

### 3.6 Sequence Conservation in Cluster 02 Proteins

The Figure 5 illustrates the comparative alignment of two additional proteins grouped in Cluster 02. In panel A, the sequence alignment demonstrates notable similarity across these proteins, despite substantial differences in polypeptide chain lengths. Panel B highlights conserved amino acid regions, suggesting the preservation of functional domains. The lower overall percentage similarity observed between these sequences, compared to Cluster 01, is likely attributable to differences in protein size. Nonetheless, the alignment reveals discrete regions of high identity, indicating evolutionary conservation of structurally or functionally relevant motifs. These conserved regions may represent binding sites or structural elements essential for the foam's protective matrix, potentially influenced by alternative splicing or post‐ translational modifications.

These results suggest that the same evolutionary mechanisms proposed for Cluster 01, such as domain retention and functional convergence, may also apply to Cluster 02. Moreover, the pattern of alignment in panel B may reflect alternative splicing events, leading to structural variants with potentially distinct regulatory or functional properties. This is further supported by the fact that bands 7, 8, and 9 on the SDS‐PAGE gel (Table 2), which correspond to these proteins, were all mapped to isoforms of a single contig or unigene derived from the RNA‐Seq transcriptome assembly of *M. spectabilis*. These findings suggest that even in the presence of structural divergence or varied molecular weights, key functional domains may be preserved, contributing to the observed similarities in the foam proteome of *M. spectabilis*.

One of the most notable findings was the identification of peptides derived from the same unigene (Gene|122372) in three distinct protein bands (B7, B8, and B9) corresponding to molecular weights of approximately 49, 45, and 40 kDa, respectively (Table 2). Although the theoretical molecular weight of the full‐length protein encoded by Gene|122372 is 50.57 kDa, the observed diversity in gel mobility suggests the occurrence of protein processing events. Among the possible mechanisms, signal peptide cleavage was predicted by the SignalP algorithm (Figure S3), which identified a canonical signal peptide at the N‐terminus with a predicted cleavage site.

After computational removal of the signal peptide, the mature protein was estimated to have a molecular weight of 48.14 kDa, consistent with the apparent mass of band B8. This supports the hypothesis that band B7 may correspond to the full‐length unprocessed form, while B8 represents the mature protein post‐cleavage. Band B9 likely corresponds to a processed or truncated form resulting from proteolytic cleavage or alternative splicing‐derived isoforms. These findings highlight the complexity of post‐translational processing among secreted foam proteins and support the idea that a single transcript can give rise to structurally related protein forms differing in mass and potentially in function.

### 3.7 Structural Modeling of Foam Proteins With AlphaFold

**Figure 6:**
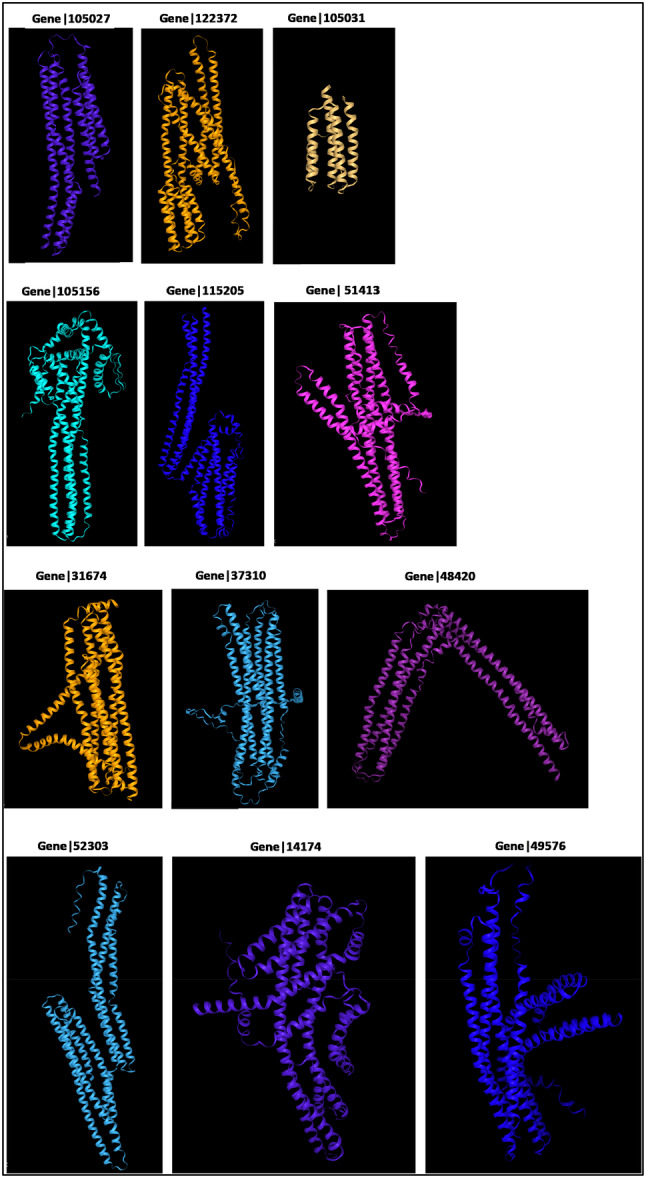
Structural models of foam proteins predicted by AlphaFold3 for *Mahanarva spectabilis* nymphs. (A) Gene|105027, (B) Gene|122372, (C) Gene|105031. Each model illustrates the predicted three‐dimensional structure of these highly abundant proteins, highlighting their shared structural features, including a prevalence of extended α‐helical secondary structures. These proteins are part of Cluster 01, characterized by high sequence similarity, and are predicted to play roles in foam stabilization, adhesion, and potentially in nymphal protection.

In the Figure 6 displays the structural models generated by AlphaFold3 for the most abundant proteins identified in the foam of *Mahanarva spectabilis* nymphs. The predicted structures exhibit a striking prevalence of extended α‐helical secondary structures across all analyzed proteins. These α‐Helices are known for providing structural rigidity and stability, which are critical for proteins in extracellular environments like the foam matrix. The recurrence of α‐ helical‐rich structures, despite differences in polypeptide length, suggests that these proteins share a conserved folding pattern that may be functionally significant. This consistent presence of α‐helices suggests a conserved folding pattern that may be critical for maintaining functional stability within the foam environment.

Despite variations in polypeptide length among the proteins, the core structural framework appears conserved, indicating selective evolutionary pressure to retain functional domains. Notably, such α‐helical architectures are commonly associated with adhesive and scaffold‐forming proteins in biological systems, including silk, collagens, and apolipophorins. Their amphipathic nature may facilitate protein–protein and protein–lipid interactions, enhancing the foam's physical integrity and water retention capacity. These structural properties could also explain the foam's resistance to degradation and its ability to adhere to both insect and plant surfaces. These structural features may contribute to the foam's ability to protect nymphs from desiccation, predators, or microbial threats, making them potential targets for pest control strategies. The structural similarity across proteins further supports earlier observations of sequence conservation, particularly within clusters identified by phylogenetic analysis.

These findings reinforce the hypothesis that the foam proteome is composed of proteins with shared structural and possibly functional features, despite sequence divergence. The presence of conserved α‐helical motifs may reflect a common role in molecular interactions, stability, or resistance to environmental stress within the extracellular foam matrix.

### 3.8 Functional Prediction of Foam Proteins Using AlphaFold and Phyre Algoritms

To further explore the structural and functional properties of the most abundant foam proteins (Table 2), predicted models were analyzed using the AlphaFold3. Table 3 summarizes the results of structural similarity searches conducted against experimentally resolved protein structures. For each protein from Table 3, AlphaFold ‐derived models were compared to known protein structures using alignment metrics such as e‐values and TM‐scores. LCMS analysis also identified several others proteins presents in the SDS‐PAGE bands (Material Supplentary – Peaks Analysis). Phyre algoritm was used to modeling and functional characterization of these proteins (Table S1).

**Table 3:** Predicted structural similarities and putative functions of foam proteins based on AlphaFold3 modeling and structural alignment.

Protein	Predict function	Score	Similar	e‐value	TM‐score
Gene|105027	Shell matrix protein	0.080	2d4c_A	0.5312	0.4606
	Talin‐1	0.041	4nqi_D	0.5273	0.4626
Gene|122372	WSC domain‐containing protein Shell matrix protein	0.055	4zwt_G 7udm_A	0.3774	0.1818
		0.054		0.3714	0.306
Gene|105031	Apolipophorin‐III	0.073	2rld_E	0.5656	0.5371
	VBS domain‐containing protein	0.020	2rld_B	0.5649	0.5469
	Talin‐2				
		0.013			
Gene|14174	S‐layer homology domain‐ containing protein SbsC_C domain‐containing protein	0.119	7w9u_A 5xsj_L	0.4836	0.2309
		0.042		0.4831	0.2223
Gene|105156	Apolipophorin	0.092	8d9o_A	0.534	0.3343
	ANK_REP_REGION domain‐	0.031	4atm_2	0.5314	0.3639
	containing protein				
Gene|115205	Outer membrane protein	0.068	3sog_2	0.4723	0.3119
	Outer membrane protein B	0.031	2fic_B	0.467	0.3064
Gene| 51413	I/LWEQ domain‐containing protein B22R‐like protein	0.018	3sog_2 4atm_2	0.5213	0.331
		0.010		0.514	0.3541
Gene|31674	Reticulocyte binding protein	0.456	8abq_C	0.4068	0.2585
	Rhoptry protein	0.061	8a1g_C	0.4007	0.2551
Gene|37310	Pv‐fam‐d protein	0.031	7n6g_S	0.3793	0.3552
	Reticulocyte binding protein 2b	0.030	7sqc_J	0.377	0.3271
Gene|48420	Reticulocyte binding protein, Tyrosine‐protein phosphatase domain‐containing protein	0.052	8gl3_A 7jh6_D	0.4908	0.3173
		0.040		0.4865	0.2915
Gene|52303	I/LWEQ domain‐containing protein C1q domain‐containing protein	0.028	4atm_2 8i7r_e	0.532	0.3648
		0.026		0.5299	0.378
Gene|49576	Secreted protein	0.028	2d4c_A	0.5031	0.3421
	(pine wood nematode)	0.025	2d4c_B	0.5011	0.3481
	hypothetical protein				

Despite the absence of functional annotation from conventional databases (GO, KEGG, KOG, Pfam), several proteins exhibited moderate to high TM‐scores (≥ 0.5) when aligned to structures with known molecular functions. These structural alignments allowed for the assignment of putative functional roles based on similarity to characterized protein domains. For each entry in the table, we report the predicted function, structural homologs, e‐values, and TM‐scores associated with the top alignments.

For example, Gene|105027 showed structural similarity to shell matrix proteins and Talin‐1, suggesting a role in extracellular matrix formation or adhesion. Gene|122372 was predicted to contain WSC domains, which are potentially involved in osmotic stress response. Shell matrix proteins (SMPs), although typically associated with biomineralization in mollusks, are known for their ability to organize stable extracellular matrices, suggesting that similar structural scaffolding functions may be co‐opted in the foam. Talin‐1 is a cytoskeletal linker protein that facilitates cell adhesion by connecting actin filaments to integrin complexes; its structural similarity to foam proteins indicates a possible role in anchoring the foam to plant surfaces or to the nymph's cuticle. The WSC domain (Wall Stress Component), commonly found in yeast and fungi, functions as a sensor of osmotic and mechanical stress, indicating that foam proteins bearing this domain may help maintain moisture balance and structural resilience under environmental challenges.

Among these, Gene|105027 likely stabilizes the foam's physical structure, ensuring it adheres to plant surfaces or the nymph, forming a robust protective barrier. Similarly, Gene|122372, with its WSC domain, may help maintain moisture balance, protecting nymphs from dehydration or excessive humidity. This analysis provides valuable structural context and supports the hypothesis of functional conservation among foam proteins, even in the absence of direct sequence homology.

Predicted structural models and functional annotations were obtained via AlphaFold analysis, allowing for putative functional classification (Table 4). Among the identified proteins, several showed high structural similarity to known matrix‐associated proteins, apolipophorins, outer membrane components, and immune‐related domains. Specifically, proteins such as Talin‐1, WSC domain‐containing proteins, Apolipophorin‐III, and S‐layer homology domain proteins were predicted with moderate to high structural confidence (TM‐ score > 0.5), indicating reliable fold assignments. The “similar” column reports the top structurally aligned natural protein entries supporting these predictions.

**Table 4:** Functional prediction and potential biological roles of abundant proteins in *M. spectabilis foam*.

Protein (Gene)	Predicted function	Potential biological role	Relevance to Nymph Life Cycle
Protein	Predict Function (Other/Unassigned)	Unknown or indirect roles.	Potentially ancillary to foam biology.
Gene|122372	transport protein (Other/Unassigned)	Unknown or indirect roles.	Potentially ancillary to foam biology.
Gene|115205	blood clotting—Fibrinogen (Coagulation/Structural)	Fibrillar networks and viscoelastic support; adhesion.	Stability/anchoring of the nymph within foam.
Gene|60882	blood clotting—FIBRINOGEN (Coagulation/Structural)	Fibrillar networks and viscoelastic support; adhesion.	Stability/anchoring of the nymph within foam.
Gene|65412	membrane protein—Virulence factor (Bacterial‐like/Toxins)	Microbiome‐linked antimicrobial and defensive actions.	Controls microbial community; deters predators/competitors.
Gene|91261	chemotaxis (Bacterial‐like/Toxins)	Microbiome‐linked antimicrobial and defensive actions.	Controls microbial community; deters predators/competitors.
Gene|124742	cell adhesion (Adhesion/Receptors/Signaling)	Adhesion to plant surfaces; receptor‐mediated regulation.	Improves attachment and persistence under environmental stress.
Gene_15229	cell adhesion (Adhesion/Receptors/Signaling)	Adhesion to plant surfaces; receptor‐mediated regulation.	Improves attachment and persistence under environmental stress.
Gene|86624	oxidoreductase (Oxidoreductases)	Phenolic cross‐linking; ROS detoxification; antimicrobial support.	Strengthens protective casing; supports stress tolerance.
Gene|87287	oxidoreductase (Oxidoreductases)	Phenolic cross‐linking; ROS detoxification; antimicrobial support.	Strengthens protective casing; supports stress tolerance.
Gene|30185	lipocalin (Transport/Lipid (Apolipoproteins/Lipocalins))	Surfactant/lipid transport; stabilization of foam films; protection from desiccation/UV.	Maintains hydrated and thermally buffered microhabitat; shields nymphs.
Gene_28090	atp synthase (Cellular Transport/Energy)	Energy production and vesicle trafficking.	Sustains continuous secretion for foam upkeep.

Functionally, the identified proteins fell into four broad categories: (i) structural matrix components (e.g., Talin‐like, shell matrix proteins), (ii) immune‐related and lipid‐binding proteins (e.g., Apolipophorin, I/LWEQ domain proteins), (iii) outer membrane or transport proteins, and (iv) hypothetical or secreted proteins of unknown function. These predicted functions suggest roles in foam cohesion, environmental buffering, innate immunity, and physiological regulation.

The putative roles assigned to these proteins suggest that their absence could severely impair the functional integrity of the foam. Hypothetically, the deletion of genes such as Gene|105027 and Gene|122372 would compromise foam stability, reduce adhesion to substrates, and impair moisture regulation. Such disruptions would likely make the nymph more vulnerable to dehydration, microbial attack, and thermal stress, ultimately increasing mortality. These proteins thus represent potential targets for interfering with foam formation, either through genetic silencing or biochemical inhibition strategies aimed at pest control.

Phyre modeling allowed integrate the updated functional assignments, for all proteins classified as unknown, into a cohesive view of the nymphal foam proteome (Table S1). The set highlights complementary axes that together explain how the foam operates as a protective, long‐lasting biointerface. First, coagulation/structural components (e.g., fibrinogen‐like matches; Gene|115205, Gene|60882) are linked to fibrillar network formation and viscoelastic support, which stabilize the bubble film and physically anchor the nymph. Oxidoreductases (e.g., laccase/SOD‐like; Gene|86624, Gene|87287) align with phenolic cross‐linking and reactive‐oxygen control, processes that can stiffen the matrix and contribute to antimicrobial resilience. Transport/lipid factors include lipocalins (e.g., Gene|30185), consistent with a protein‐lipid surfactant system that lowers interfacial tension, limits water loss and provides optical/antioxidant shielding. Adhesion and receptor‐type proteins (e.g., discoidin/cadherin‐like; Gene|124742, Gene_15229) plausibly reinforce attachment of the foam to plant surfaces and help it persist under environmental stress. Bacterial‐like/toxin signatures (e.g., virulence/chemotaxis; Gene|65412, Gene|91261) point to a microbiome contribution, offering potential antimicrobial or deterrent effects that help manage the foam's microbial community. Cellular transport/energy entries (e.g., ATP‐synthase; Gene_28090) likely reflect the secretory and energetic demands of sustained foam production and upkeep. Finally, a minority of proteins remain “other/unassigned,” acknowledging unknown or indirect roles. Collectively, these classes depict the foam as an emergent composite‐surfactant, structural, redox‐active and microbially fortified‐tailored to maintain a hydrated, thermally buffered and defended microhabitat for *Mahanarva spectabilis* nymphs.

**Figure 7:**
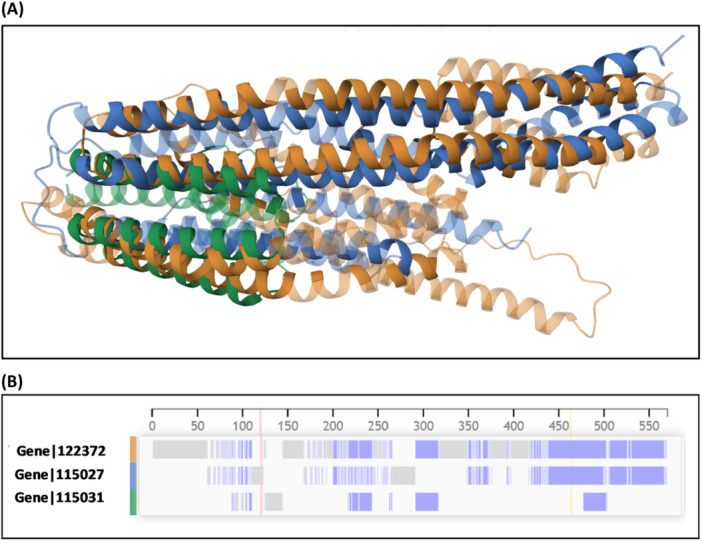
Structural alignment of three abundant foam proteins from *Mahanarva spectabilis* (Gene|105027, Gene|122372, and Gene|105031) identified in Cluster 01. (A) Superimposed 3D models generated by AlphaFold and aligned using the PDB structural alignment tool, showing a high degree of overlap despite differences in polypeptide length (14–50 kDa). (B) Diagram of conserved structural regions across the three proteins, indicating retention of α‐helical domains and suggesting functional conservation potentially related to foam stability or host–microbe interactions.

Structural alignments were performed for three proteins identified in Cluster 01: Gene|105027 ( ~ 34 kDa), Gene|122372 ( ~ 50 kDa), and Gene|105031 ( ~ 14 kDa), all of which exhibited high sequence similarity. Despite their varying molecular weights, the structural modeling and alignment using AlphaFold and the PDB structural alignment tool revealed a remarkably high degree of overlap across extended regions of their peptide chains. Panel A of Figure 7 shows the superimposed three‐dimensional structures, indicating a consistent α‐helical core among the proteins. Panel B presents the pairwise alignment diagram highlighting structurally conserved domains. These findings suggest that, although differing in polypeptide length, the proteins maintain conserved structural motifs that may be essential for shared or complementary biological functions in the foam matrix. The observed structural conservation supports the hypothesis that these proteins have evolved to retain a core functionality, potentially contributing to foam stability, microbial defense, or interaction with host‐derived molecules.

**Figure 8:**
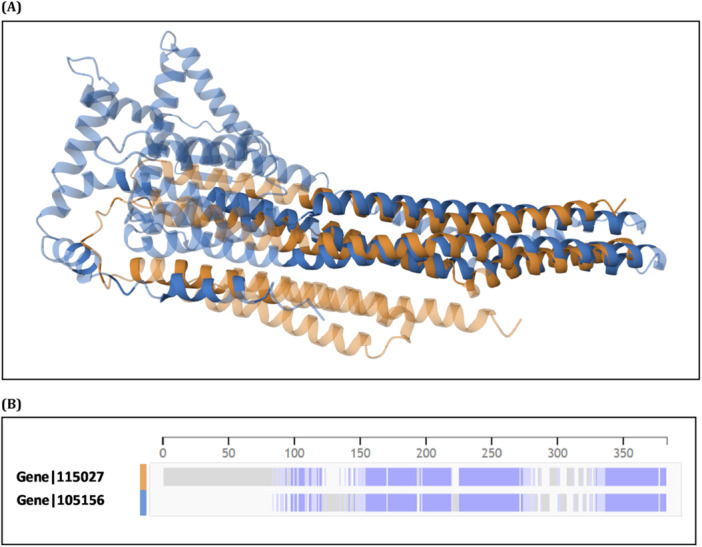
Structural alignment of two abundant foam proteins from *Mahanarva spectabilis* (Gene|105027 from Cluster 01 and Gene|105156 from Cluster 03), which exhibit lower sequence similarity. (A) Superimposed 3D models generated by AlphaFold and aligned using the PDB structural alignment tool. (B) Diagram highlighting.

To evaluate structural conservation in cases of low sequence similarity, models of two abundant foam proteins, Gene|105027 (from Cluster 01) and Gene|105156 (from Cluster 03), were aligned using AlphaFold predictions and the PDB alignment algorithm. Although these proteins differ in molecular size and show less amino acid identity than others within the cluster, the 3D structural superposition revealed conserved folds along extensive portions of their chains. Figure 8A illustrates the superimposed structures, where overlapping regions are evident along α‐helical segments. Figure 8B presents the residue‐level diagram confirming areas of structural correspondence. These findings suggest that even proteins with divergent primary sequences may retain similar tertiary architectures, supporting the hypothesis that structural conservation, rather than strict sequence identity, underlies shared functional roles in the foam microenvironment.

### 3.9 AlphaFold‐Multimer Predicts Stable Dimer Formation of the Most Abundant Protein in *Mahanarva spectabilis* Foam

**Figure 9:**
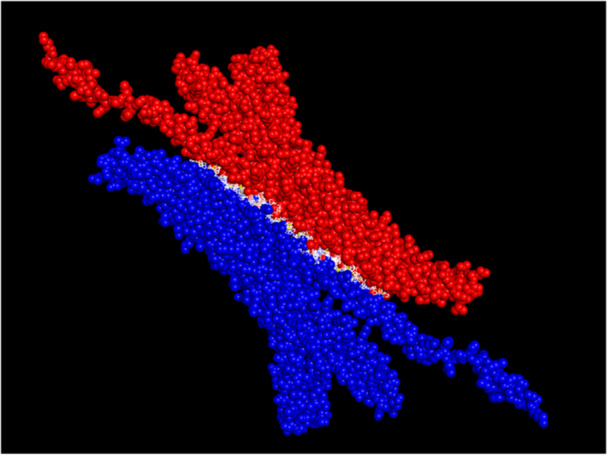
Predicted dimeric structure of the foam protein encoded by *Gene* | *122372* based on AlphaFold‐Multimer modeling. The most reliable model (ranked 001) shows two monomers interacting through a well‐defined interface, with a predicted interface TM‐score (ipTM) of 0.89 and a predicted TM‐score (pTM) of 0.86, indicating high confidence in the structural model and interaction. The dimer displays extended α‐helical regions and a stable interface, supporting its potential role in forming supramolecular networks within the foam matrix of *Mahanarva spectabilis*.

Dimerization of the protein associated with the extracellular foam of *Mahanarva spectabilis* was predicted using the AlphaFold‐Multimer model via the ColabFold platform. The most reliable model (Rank 001) was selected based on internal confidence metrics (Figure 9). This model presented a predicted interface TM‐score (ipTM) of approximately 0.89 and a predicted TM‐score (pTM) of 0.86, indicating high structural reliability and strong confidence in the interaction between the two monomers. Dimerization may enhance protein stability, create binding sites for ligands, or facilitate the formation of higher‐order structures, potentially contributing to the foam's cohesive matrix and protective functions. These values suggest a biologically plausible dimeric conformation with a well‐defined interface.

## 4 Discussion

In addition to structural and biochemical analyses, a deeper understanding of the ecological and evolutionary context of foam production in *Mahanarva spectabilis* provides new perspectives on its role in host‐plant adaptation. In our proteomic analyses, we identified multifunctional proteins in the foam, such as apolipophorin‐III, C1q domain‐containing proteins, and WSC‐domain proteins, which may contribute not only to the foam's physical structure but also to immune defense, microbial interaction, and osmotic regulation. These findings suggest that the foam acts as a dynamic biochemical interface rather than a passive barrier, reinforcing its role in nymph survival and environmental resilience.

Moreover, the identification of adhesive and matrix‐forming domains, such as ankyrin repeats, S‐layer homology (SLH), and I/LWEQ motifs, highlights a high degree of structural specialization. AlphaFold3‐based structural predictions suggest these proteins are capable of dimerization and form supramolecular scaffolds critical for foam cohesion. These suprastructures are likely shaped by evolutionary pressures to ensure foam stability and adhesion under stress, including desiccation and microbial threats. Such specialization is not restricted *to M. spectabilis*. Comparative studies on other Cercopidae species, like *Aphrophora alni*, reveal similar protein‐rich foams with viscoelastic properties and environmental adhesiveness. These parallels strengthen the hypothesis that proteinaceous foams across hemipteran species are the result of convergent evolution toward extracellular matrices with protective, structural, and regulatory functions.

Therefore, characterizing the foam proteome at both compositional and structural levels uncovers potential targets for pest management. Proteins with conserved domains related to microbial interaction and surface adhesion could be disrupted using biocontrol agents such as *Metarhizium anisopliae*, enhancing their efficacy. These insights bridge molecular entomology with sustainable agricultural practices, reinforcing the need to explore insect‐derived extracellular matrices as both evolutionary adaptations and applied pest control targets.

In addition to structural and biochemical analyses, it is crucial to contextualize the ecological significance of foam production by *Mahanarva spectabilis* within the broader spectrum of insect‐plant interactions and pest management. Previous literature has emphasized the adaptive nature of foam not only as a protective barrier but also as a multifunctional matrix capable of microbial regulation and thermal insulation (Tonelli et al. 2020; Hoch et al. 2024). The compositional complexity of this foam, enriched in proteins, surfactants, and polysaccharides, supports its roles beyond passive protection, acting as a dynamic interface with the environment.

The molecular characterization of the foam produced by *Mahanarva spectabilis* nymphs provides valuable insights into the biochemical and physiological adaptations that support the insect's survival during its early developmental stages. This pest causes significant economic losses in tropical forage production, with millions of dollars in damages annually due to reduced pasture yields and increased management costs (Oliveira et al. 2014; Espitia Buitrago et al. 2022). Targeting the nymphal phase is particularly strategic for pest management, as it may lead to a significant reduction in both the immediate damage caused by nymphs and the subsequent population density of adult insects. This is especially relevant considering that adult *M. spectabilis* individuals also feed on forage grasses, contributing to plant stress and yield loss in pastures (López 2012). Controlling nymphs before they reach maturity can therefore interrupt the pest's life cycle, decreasing the reproductive output and the size of future generations (Orozco‐Restrepo et al. 2017). Such population‐level effects have important implications for minimizing long‐term infestations and associated agricultural losses. By interfering with the foam's protective functions (physical, biochemical, or microbiological), it becomes possible to compromise nymphal survival and increase their vulnerability to environmental stressors and biological control agents (Mascarin et al. 2019). Thus, the identification of abundant proteins in the foam not only enhances our understanding of insect‐ host interactions but also offers potential molecular targets for the development of novel, sustainable control strategies aimed at reducing the economic impact of *M. spectabilis* in forage production systems.

Although no significant differences were observed in the total protein concentration of the foam produced by *Mahanarva spectabilis* nymphs across different host plant genotypes, the proteomic analysis revealed important quantitative differences in protein abundance profiles. Notably, some of the highly abundant proteins were found to be down‐regulated when nymphs fed on resistant/moderately resistant grass genotypes. These specific changes, although not reflected in overall protein content, suggest a potential disruption in the composition and functional properties of the foam (Silva 2012). Such molecular alterations may compromise the protective functions of the foam, including hydration, microbial defense, and thermal buffering, which are critical for nymphal development under natural environmental conditions. In resistant/moderately resistant hosts, the down‐regulation of key foam proteins may represent a plant‐induced stress response or an indirect effect of limited nutritional quality, ultimately affecting nymph survival in the field. Therefore, proteomic variations in the foam may contribute to the resistance/moderately resistance phenotype and have broader ecological implications, offering valuable insights for breeding resistant grass varieties. These findings underscore the importance of characterizing not only the quantity but also the quality of insect secretions in plant–insect interaction studies.

Interestingly, when individual protein bands excised from the 1D SDS‐PAGE gel were subjected to LC‐MS/MS analysis, the resulting peptide spectra mapped to multiple distinct unigenes derived from the *M. spectabilis* transcriptome. This was observed even in bands corresponding to proteins with markedly different molecular weights. For instance, peptides matching Gene|122372 were detected in bands B7, B8, and B9, despite their varying apparent molecular weights. These findings suggest the existence of protein isoforms or distinct proteins sharing highly conserved sequence regions. Such cases likely reflect the presence of gene families encoding paralogous proteins with high sequence similarity, or alternatively, post‐ translational processing or degradation products retaining common peptide sequences. This complexity indicates that the foam contains a diverse mixture of proteins with potentially overlapping functions, crucial for its multifaceted protective roles.

This hypothesis was further supported by pairwise sequence alignments and structural comparisons. These analyses confirmed that different unigenes encode proteins with significant sequence identity, especially in functionally conserved domains. From an evolutionary perspective, the expansion of gene families through duplication followed by divergence is a well‐established mechanism in insects, particularly for genes involved in interactions with the external environment, such as salivary proteins, digestive enzymes, or secreted factors. Several studies have reported similar patterns in hemipteran and lepidopteran species, where multigene families have diversified to support host adaptation, niche specialization, or immune evasion (Battistuzzi et al. 2018; Heidel‐Fischer and Vogel 2015; Hogenhout and Bos 2011). In this context, the presence of related but distinct proteins in the foam of *M. spectabilis* may reflect a lineage‐specific expansion of secreted protein families that contribute to the protective or adhesive properties of the foam. These findings underscore the importance of combining proteomic and transcriptomic data to fully resolve the complexity of insect extracellular matrices and their evolutionary underpinnings.

In addition to the conservation observed at the level of primary amino acid sequence, our analyses revealed a remarkable preservation of structural motifs and functional domains among several of the most abundant proteins identified in the foam. Structural alignments of AlphaFold3‐predicted models demonstrated that, despite originating from distinct unigenes, several proteins share similar three‐dimensional folds and possess conserved domain architectures (Table 3; Figure 7). These include apolipophorin‐related proteins, shell matrix‐ like proteins, and I/LWEQ domain‐containing proteins, among others.

The predicted structural similarity was further supported by TM‐scores above 0.5 for many protein pairs, indicating shared topological features. For example, both Gene|105027 and Gene|122372 showed structural homology to shell matrix proteins, while Gene|105031 and Gene|105156 were associated with apolipophorin and Talin‐related folds. This suggests that these proteins perform analogous biological functions within the foam matrix, such as lipid transport, adhesion, or extracellular stabilization, likely driven by evolutionary pressures to maintain foam integrity (Niere et al. 2001).

The convergence of structure and function across multiple gene products likely reflects an evolutionary process of gene duplication followed by subfunctionalization or neofunctionalization, a mechanism widely documented in insects, particularly for gene families involved in secretion and environmental interactions. Indeed, the expansion and diversification of secreted proteins, including salivary effectors and foam‐associated proteins, have been reported in several hemipteran lineages (Boulain et al. 2018). Such conservation may reflect strong selective pressures to maintain functional redundancy or robustness in the extracellular defenses of nymphs, especially given the protective role of the foam against abiotic stress and predation. Together, these findings reinforce the hypothesis that *M. spectabilis* secretes a specialized set of structurally conserved proteins, potentially derived from a common ancestral repertoire, optimized through evolution to support nymphal survival in a hostile environment.

The presence of diverse proteins in the foam produced by *M. spectabilis* nymphs suggests that this extracellular matrix serves more than a physical protective role. Several of the identified proteins, such as Apolipophorin‐III and C1q domain‐containing proteins, are known to participate in innate immune responses in insects, particularly through lipid transport and microbial recognition (Whitten et al. 2004; Niere et al. 2001; Sun 2012). These proteins may protect nymphs from microbial pathogens, enhancing the foam's role as a defensive barrier. The detection of Talin‐1‐like and shell matrix proteins may indicate involvement in stabilizing the foam's structure, possibly enhancing adherence to plant surfaces or facilitating anchorage of the insect within the foam (Weis and Nelson 2006; Baster et al. 2025). This structural stability could be targeted to disrupt the foam's protective function, reducing nymph survival.

Moreover, the identification of outer membrane‐like proteins and WSC domain‐ containing proteins points to a potential role in osmoregulation or microbial interaction within the foam environment. Proteins with WSC domains, such as WSC1I in *Beauveria bassiana*, function as sensors of osmotic, oxidative, and cell wall stress via the Hog1/MAPK pathway (Jiang 2018), and the family of WSC proteins in *Saccharomyces cerevisiae* act as mechanosensors for cell wall integrity under various stress conditions (Levin 2011; Dupres et al. 2009).

These proteins may maintain osmotic balance, protecting nymphs from dehydration or excessive moisture. Although there is no direct evidence that *Mahanarva spectabilis* expresses proteins homologous to rhoptry or reticulocyte‐binding proteins (RBPs), these proteins are well‐characterized in apicomplexan parasites, where they mediate host cell invasion (e.g., RAPs, RONs, and RBLs in Plasmodium) (Boucher and Bosch 2015). Therefore, we hypothesize that if such proteins are indeed present in *M. spectabilis*, they may act through analogous mechanisms, such as molecular mimicry or modulation of the environmental microbiota, as part of its developmental strategy. This hypothesis, however, requires validation through transcriptomic, proteomic, and structural analyses.

This suggests the foam could modulate microbial communities, potentially influencing nymph health or plant interactions. Altogether, the functional prediction of foam proteins reinforces the view that this secretion plays an integrated role in nymph survival, encompassing structural integrity, immunological protection, and possible microbiota modulation.

The predicted dimerization of the most abundant foam protein in *Mahanarva spectabilis*, supported by high‐confidence AlphaFold‐Multimer scores, strengthens the hypothesis that this protein participates in network formation within the extracellular foam matrix. This structural arrangement is consistent with functions associated with matrix stabilization, adhesion, and physical resilience. Notably, the predicted dimeric interface may facilitate multimeric assembly, creating a scaffold capable of sustaining foam structure and promoting its adhesion to insect and plant surfaces. This dimerization is particularly significant, as it may enhance the foam's viscoelastic properties, crucial for maintaining stability under environmental stress (Hoch et al. 2024).

This hypothesis is reinforced by two additional observations. First, the most abundant foam proteins consistently displayed long, repetitive α‐helical regions, a feature commonly associated with structural and adhesive proteins in biological systems. Similar α‐helical motifs are well‐characterized in spider silk, apolipophorins, and extracellular matrix proteins that form ordered fibrillar or mesh‐like assemblies. Second, structural modeling and alignment revealed that multiple proteins shared conserved folds and functional domains (such as WSC, S‐layer homology, and apolipophorin‐related regions) across different unigenes, suggesting evolutionary conservation and redundancy in adhesive or scaffold‐forming roles.

Corroborating this, Hoch et al. (2024) demonstrated that spittlebug foam from *Aphrophora alni* exhibits adhesive properties that depend on hydration level and biochemical composition, including mucopolysaccharides and glycoproteins. The foam adhered to various surfaces and formed stable lamellar and filamentous structures, indicating the presence of viscoelastic and cohesive elements, likely driven by protein‐protein interactions and polymeric networks. Notably, pulled filaments and bead‐on‐a‐string structures observed under tension in the foam (Hoch et al. 2024) are hallmark features of materials containing extended, aligned α‐helices and amphiphilic domains. Thus, our findings suggest that the dimeric, α‐ helical‐rich proteins secreted in *M. spectabilis* foam may represent specialized, evolutionarily adapted matrix‐forming components. Their structural features and conserved domains imply functional convergence toward building a cohesive and adhesive foam scaffold, critical for nymph protection and environmental stability.

Among the structurally conserved proteins identified in the foam of *Mahanarva spectabilis*, several exhibited specific functional domains that further support their potential roles in adhesion, matrix stabilization, and extracellular interactions. Notably, proteins encoded by Gene|122372 were predicted to contain a WSC (Wall Stress Component) domain, typically found in fungal and plant proteins involved in cell wall integrity and adhesion to external surfaces. Although rarely described in insects, the presence of a WSC‐like domain suggests a convergent adaptation to extracellular stabilization or mechanical anchoring, possibly within the foam matrix. This domain could be targeted to disrupt foam adhesion, offering a novel pest control approach.

Similarly, apolipophorin‐III‐like proteins (Gene|105031 and Gene|105156) were identified with high‐confidence structural similarity to Talin and VBS domain‐containing proteins. Apolipophorins in insects are multifunctional, often associated with lipid transport, immune response, and extracellular vesicle formation. Their amphipathic and α‐helical nature enables them to interact with lipid–water interfaces, suggesting a surfactant‐like function in foam stabilization, potentially modulating bubble integrity or surface tension. These properties could be exploited to develop agents that destabilize the foam's structure.

Another notable domain detected was the ANK_REP_REGION (ankyrin repeat region), which mediates protein–protein interactions and is commonly found in proteins involved in scaffolding and structural organization. Its presence in secreted proteins suggests a role in multimeric assembly or interaction with other foam constituents, contributing to the cohesive architecture of the foam.

Proteins bearing the S‐layer homology (SLH) domain (Gene|14174) (typically involved in bacterial surface layer formation may represent horizontal gene transfer‐derived or convergently evolved elements adapted for extracellular assembly. These domains are known to mediate anchoring to polysaccharide or glycoprotein matrices, aligning with the known glyco‐rich nature of spittlebug foam. The I/LWEQ domain found in proteins such as Gene|52303 and Gene|51413 is classically associated with cytoskeletal and membrane‐associated proteins involved in actin binding and cellular adhesion. Its presence in extracellular proteins of the foam may reflect a novel functional adaptation, possibly mediating elastic or viscoelastic interactions across the foam lamellae.

Collectively, these domain architectures reinforce the hypothesis that the foam proteins of *M. spectabilis* are evolutionarily adapted for extracellular functionality, combining features of adhesion, elasticity, and matrix organization. Their structural and functional convergence echoes the complexity and multifunctionality observed in other biological foams, such as frog nest proteins and spittlebug secretions in *Aphrophora* species. These observations point to a sophisticated molecular toolkit underlying the protective and adhesive properties of the foam, with possible biomimetic implications.

## 5 Conclusion

Here, we present an integrated proteomic and structural characterization of the foam produced by *Mahanarva spectabilis* nymphs, revealing a set of highly abundant extracellular proteins with potential roles in foam stabilization, adhesion, and nymphal protection. Despite the absence of significant differences in total protein concentration across different host plant genotypes, key proteins were down‐regulated in resistant/moderately resistant hosts, suggesting a possible impairment of foam integrity and a reduction in nymph survival under natural conditions.

Proteomic and structural analyses revealed that several abundant proteins, though encoded by distinct unigenes, share highly conserved sequence motifs, domain architectures, and structural folds. These findings were further supported by AlphaFold‐based structural predictions, which indicated that multiple proteins possess adhesive or matrix‐forming domains, including WSC, apolipophorin, S‐layer homology, and ankyrin repeat motifs. The predominance of extended α‐helices and the predicted capacity for dimerization in the most abundant foam protein reinforce the hypothesis that these molecules contribute to the formation of supramolecular networks essential for the physical resilience of the foam.

Taken together, our results suggest that the foam is a structurally organized and biochemically complex extracellular matrix shaped by evolution to ensure the survival of the nymph in hostile environmental conditions. The identification of conserved adhesive domains and matrix‐related motifs opens new perspectives for exploring biomimetic materials and for the development of novel strategies targeting the structural integrity of the foam as a means of controlling *M. spectabilis* populations in forage systems.

These networks likely enable the foam to shield nymphs from environmental stressors, such as desiccation and predation, making them prime targets for pest control strategies. Collectively, these results demonstrate that the foam is a sophisticated, biochemically complex extracellular matrix, shaped by evolutionary pressures to ensure nymph survival in challenging environments. The identification of conserved adhesive and matrix‐related domains not only deepens our understanding of insect‐plant interactions but also opens new avenues for practical applications. Specifically, targeting these proteins could lead to innovative pest control methods, such as developing inhibitors or biological agents like *Metarhizium anisopliae* to disrupt foam stability, thereby reducing nymph survival and mitigating the economic impact of *M. spectabilis* on tropical forage systems. Moreover, the unique structural properties of these proteins offer exciting opportunities for biomimetic research, potentially inspiring the development of novel adhesives or protective coatings with applications in agriculture and beyond. This study fills a critical gap in our knowledge of *M. spectabilis* biology, providing a foundation for future research into sustainable pest management and bioinspired materials.

Further studies could validate these findings through functional assays or field trials, paving the way for environmentally friendly solutions to manage this significant agricultural pest.

## Author Contribution

**Angelo José Rinaldi:** methodology, formal analysis, writing – review and editing, writing – original draft, software, investigation. **Miss Monique da Silva Bonjour:** formal analysis, writing – original draft, writing – review and editing, methodology, software. **Ian de Paula Alves Pinto:** formal analysis, methodology. **Gabriely Teixeira Bhering Faria:** formal analysis, methodology, software. **Lucas Leal Lima:** formal analysis, methodology, software. **Marcela Chellini Pereira:** formal analysis, methodology. **Alexander Machado Auad:** conceptualization, writing –original draft, writing – review and editing, projectadministration, methodology, fundingacquisition, investigation. **Jorge Fernando Pereira:** methodology, conceptualization, writing – original draft, writing – review and editing, projectadministration, fundingacquisition, investigation. **Maria Goreti Almeida Oliveira:** conceptualization, methodology, writing – review and editing, writing – original draft, project administration, supervision, funding acquisition, investigation. **Humberto Josué de Oliveira Ramos:** conceptualization, formal analysis, data curation, writing – original draft, writing – review and editing, supervision, methodology, software, funding acquisition, project administration.

## Conflicts of Interest

The authors declare no conflicts of interest.

## Supporting information


**Figure S1:** Comparison of protein profiles from the extracellular foam produced by *Mahanarva spectabilis* nymphs feeding on four different forage grass genotypes. **Figure S2:** Heatmap showing pairwise amino acid identity among these proteins. **Figure S3:** Signal peptide prediction for the protein encoded by *Gene|122372* using the Signal P 6.0 algorithm.
