# Supplementary material for: Molecular and Structural Characterization of Foam Proteins from Mahanarva spectabilis (Distant, 1909) (Hemiptera: Cercopidae) Nymphs Reveals Adaptive Features and Potential Targets for Pest Control
Source: Arch Insect Biochem Physiol. 2026 Feb 17;121(2):e70130. doi: 10.1002/arch.70130 (PMC12911473; doi:10.1002/arch.70130)
Supplement: Supplementary file 1 — Figure S1: Comparison of protein profiles from the extracellular foam produced by Mahanarva spectabilis nymphs feeding on four different forage grass genotypes. Figure S2: Heatmap showing pairwise amino acid identity among these proteins. Figure S3: Signal peptide prediction for the protein encoded by Gene|122372 using the Signal P 6.0 algorithm. [file ARCH-121-e70130-s001.docx]

**Supplementary Material:**

**Figure S1:** Comparison of protein profiles from the extracellular foam produced by *Mahanarva spectabilis* nymphs feeding on four different forage grass genotypes. Proteins were separated by one-dimensional SDS-PAGE and visualized by Coomassie staining. Each lane represents a biological replicate corresponding to one host plant genotype.


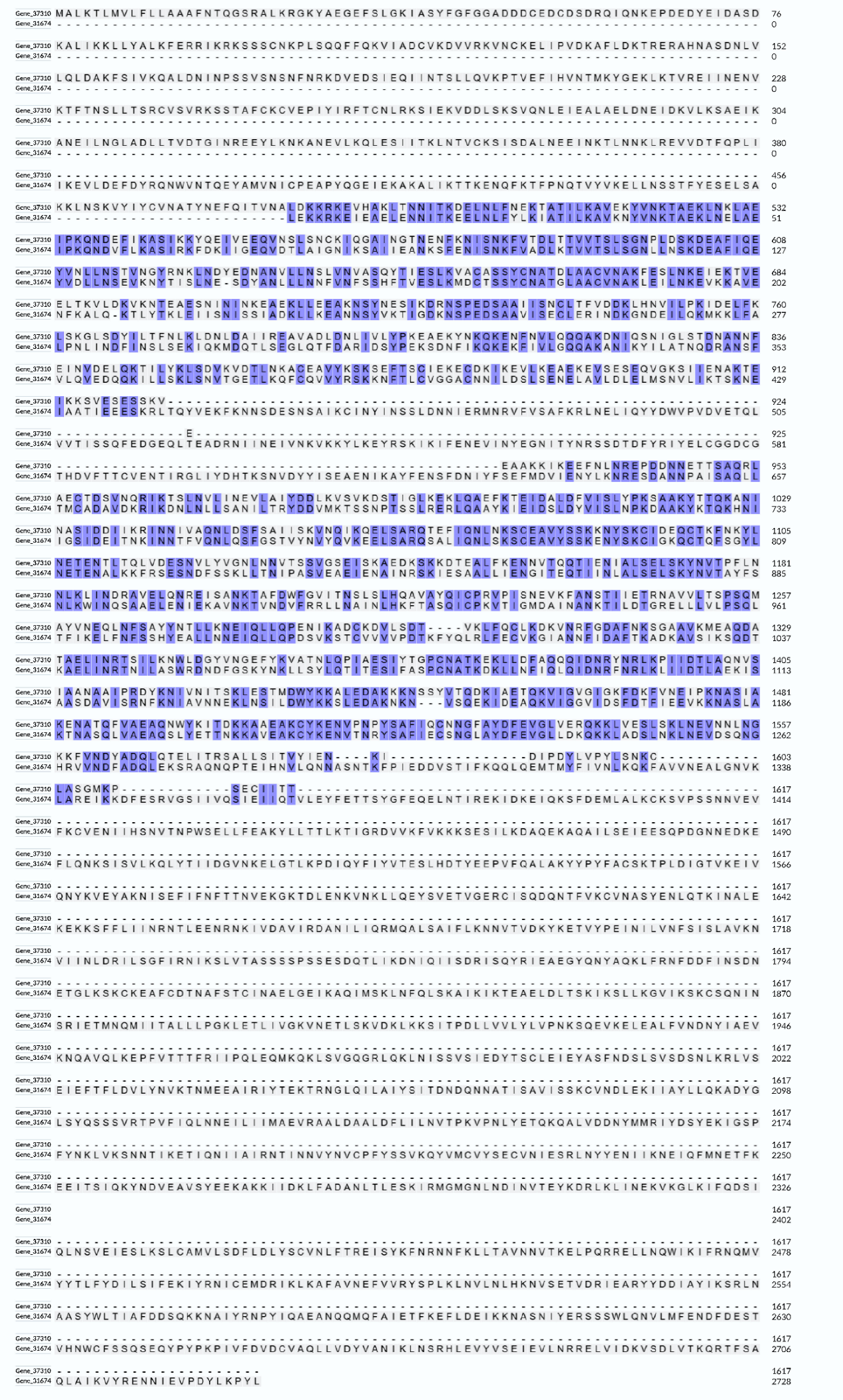


**Figure S2:** Heatmap showing pairwise amino acid identity among these proteins. Sequence alignment was performed using the UniProt alignment tool (Clustal Omega algorithm).


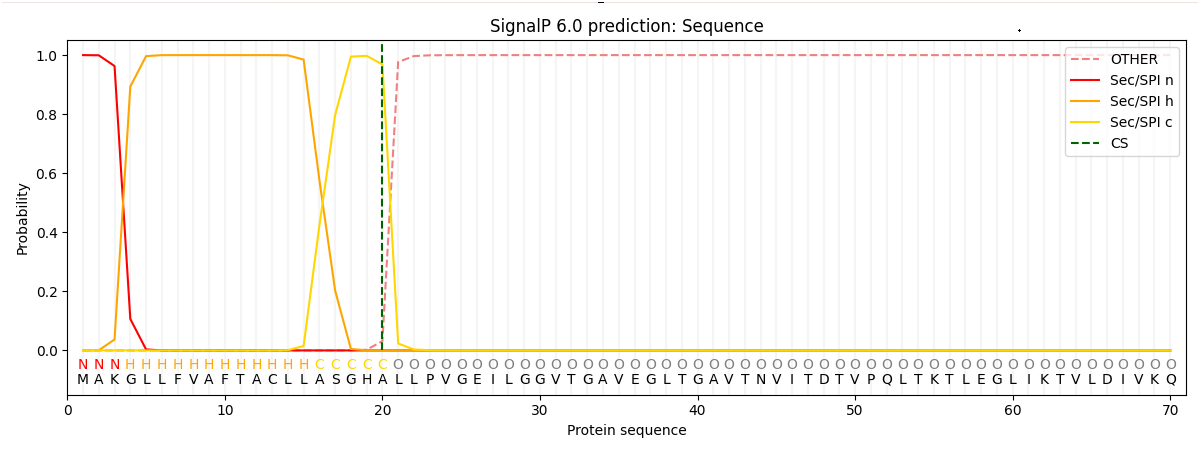


**Figure S3:** Signal peptide prediction for the protein encoded by Gene|122372 using the SignalP 6.0 algorithm. The analysis revealed a canonical N-terminal signal peptide with a predicted cleavage site between amino acid positions 22 and 23. The presence of this signal sequence indicates that the protein is secreted via the classical secretion pathway. The mature form of the protein, following signal peptide removal, is predicted to have a molecular weight of approximately 48.14 kDa, compared to the full-length sequence of 50.57 kDa. This processing step likely accounts for the differential migration observed in SDS-PAGE protein bands B7, B8, and B9.

**Table S1:**Predicted structural similarities and putative functions of foam proteins based on Phyre 2.2 modeling and structural alignment.

| **Protein** | **Predicted Function** | **Similarity** | | **% confidence** | | **% coverage** | |
| --- | --- | --- | --- | --- | --- | --- | --- |
|  |  | **Template** | **PDB Molecule** |  |  |  |  |
| Gene\|122372 | transport protein | c3s84B_ | Apolipoprotein A-IV | 86.7% | 47% | |  |
| Gene\|37310 | metal transport | c1ji5A_ | Dlp-1 | 18.7% | 7% | |  |
| Gene\|105156 | signaling protein | c7zawA_ | Glypican-3 | 14.5% | 6% | |  |
| Gene\|105027 | viral protein | c3mawA_ | Fusion Glycoprotein F0 | 15.5% | 17% | |  |
| Gene\|14174 | lipid binding protein | c9e9rA_ | Apolipoprotein B 100 | 57.9% | 44% | |  |
| Gene\|55109 | lipid binding protein | c1eq1A_ | Apolipophorin-III | 67.2% | 4% | |  |
| Gene\|105031 | viral protein | c2xz3A_ | Maltose ABC transporter periplasmic protein, envelope glycoprotein | 80.9% | 64% | |  |
| Gene\|49576 | lipid transport | c2lemA_ | Apolipoprotein A-1 | 35.4% | 8% | |  |
| Gene\|32429 | membrane protein | c8fedD_ | Virulence factor mce family protein | 56.6% | 82% | |  |
| Gene\|115205 | blood clotting - Fibrinogen | c1m1jB_ | Fibrionogen beta chain | 8.4% | 6% | |  |
| Gene\|82896 | toxin | c6w08B_ | Motility Associeted killing factor E | 59.0% | 24% | |  |
| Gene\|60882 | blood clotting - FIBRINOGEN | c1ei3C_ | Fibrinogen | 7.9% | 7% | |  |
| Gene\|114104 | toxin | c6gy6B_ | XaxB | 94.5% | 41% | |  |
| Gene\|86182 | protein fibril | c6vy1A_ | Prefoldin subunit alpha 2 | 75.8% | 22% | |  |
| Gene\|48420 | motor protein | c8q6tH_ | Myosin-7 | 87.1% | 100% | |  |
| Gene\|28910 | motor protein | c6zyxC_ | Dynein light chain | 80.7% | 16% | |  |
| Gene\|63546 | plant protein - Microtubule binding protein | c8y9rA_ | Microtubele binding protein | 18.0% | 76% | |  |
| Gene\|52303 | blood clotting - fibrinogen | c1ei3B_ | fibrinogen | 47.1% | 8% | |  |
| Gene\|103375 | viral protein | c8ixkE_ | Head virion protein G6P | 12.3% | 64% | |  |
| Gene\|1606 | signaling protein | c1dg3A_ | PROTEIN (INTERFERON-INDUCED-GUANYLATE-BINDING PROTEIN 1) | 7.7% | 26% | |  |
| Gene\|107961 | hydrolase inhibitor | c3ozqA_ | Serpin48 | 100.0% | 92% | |  |
| Gene\|91256 | transport protein | c3s84B_ | Apolipoprotein A-IV | 86.0% | 29% | |  |
| Gene\|41141 | transferase | c3kczA_ | Poly[ADP-ribose] polymerase 2 | 11.0% | 14% | |  |
| Gene\|25201 | membrane protein | c2x43A_ | sherp | 15.6% | 20% | |  |
| Gene\|29705 | viral protein  - Spike protein S2 | c6x45E_ | Spike protein s2 | 31.8% | 34% | |  |
| Gene\|103377 | toxin | c3eb7A_ | Insecticidal Delta-Endotoxin Cry8Ea1 | 54.9% | 78% | |  |
| Gene\|31674 | blood clotting - fibrinogen | c1ei3B_ | fibrinogen | 35.4% | 4% | |  |
| Gene\|51413 | lipid binding protein | c9e9rA_ | Apolipoprotein B 100 | 45.3% | 25% | |  |
| Gene\|65412 | membrane protein - Virulence factor | c8fedA_ | Virulence factor Mce family protein | 37.2% | 100% | |  |
| Gene\|25773 | membrane protein | c6ek8A_ | YaxB | 22.1% | 17% | |  |
| Gene\|65410 | viral protein | c4njlA_ | S protein | 37.0% | 70% | |  |
| Gene\|100644 | viral protein | c5w82D_ | Protein Delta | 25.1% | 33% | |  |
| Gene\|31020 | transport protein/chaperone | c2p58A_ | Putative type III secretion protein YscE | 53.8% | 14% | |  |
| Gene\|13847 | hydrolase | c7mp4A_ | Carboxylesterase-24 | 100.0% | 90% | |  |
| Gene\|124738 | signaling protein | c4xbmB_ | Delta-like protein 1 | 99.1% | 34% | |  |
| Gene\|127572 | hydrolase/hydrolase inhibitor | c4bd9B_ | Carboxypeptidase inhibitor | 99.2% | 37% | |  |
| Gene\|9596 | lipid binding protein | c1eq1A_ | APOLIPOPHORIN-II | 87.7% | 11% | |  |
| Gene\|8964 | hydrolase/hydrolase inhibitor | c4gi3B_ | Greglin | 90.2% | 22% | |  |
| Gene\|73191 | metal transport | c4egwA_ | Magnesium transport protein CorA | 53.9% | 15% | |  |
| Gene\|80872 | signaling protein | c4xbmB_ | Delta-like protein 1 | 96.7% | 34% | |  |
| Gene\|91261 | chemotaxis | c2ch7B_ | Methyl-accepting chemotaxis protein | 92.5% | 58% | |  |
| Gene\|16112 | virus | c7bzuE_ | KRM1 | 98.5% | 22% | |  |
| Gene\|27604 | lyase | c4rv3A_ | 1-Phosphatidylinositol phosphodiesterase | 100.0% | 75% | |  |
| Gene\|103088 | structural protein | c6fpzA_ | Inter-alpha-trypsin inhibitor heavy chain H1 | 100.0% | 69% | |  |
| Gene\|124742 | cell adhesion | c4d90A_ | EFG-like repeat and discoidin 1-like domain-containing protein 3 | 97.7% | 77% | |  |
| Gene\|11151 | lipid binding protein | c5e7xA_ | Cell wall antigen | 6.5% | 77% | |  |
| Gene\|49975 | transcription | c8qr1E_ | Actin, cytoplasmic 1, N-terminally processed | 100.0% | 99% | |  |
| Gene\|61710 | transcription | c8qr1E_ | Actin, cytoplasmic 1, N-terminally processed | 100.0% | 99% | |  |
| Gene\|106927 | lyase | c2or2A_ | 1-Phosphatidylinositol phosphodiesterase | 100.0% | 77% | |  |
| Gene\|51166 | P  lant protein - Microtubule binding protein | c8y9rA_ | Microtubule binding protein | 8.9% | 67% | |  |
| Gene\|86624 | oxidoreductase | c8r7iA_ | Laccase BP 76 | 100.0% | 92% | |  |
| Gene\|105416 | structural protein | c8tidW_ | WD40 domain protein | 98.5% | 93% | |  |
| Gene\|105105 | transport protein | c2hzrA_ | Apolipoprotein D | 99.0% | 74% | |  |
| Gene\|87287 | oxidoreductase | c8jd8D_ | Superoxide dismutase [Cu-Zn] | 100.0% | 81% | |  |
| Gene\|69767 | signaling protein | c7x6fD_ | Quorum-sensing regulator protein G | 7.4% | 6% | |  |
| Gene\|102840 | Prostaglandin-H2 D-isomerase | c4orrA_ | Prostaglandin-H2 D-isomerase | 97.4% | 71% | |  |
| Gene\|116606 | hydrolase | c7mp4A_ | Carboxylesterase-24 | 100.0% | 96% | |  |
| Gene\|120316 | transcription | c7emfC_ | Mediator of RNA polymerase II transcription subunit 4 | 18.5% | 17% | |  |
| Gene\|48087 | isomerase/dna | c5eixA_ | DNA topoisomerase 4 subunit B, DNA topoisomerase 4 subunit A | 31.2% | 15% | |  |
| Gene\|100642 | oxidoreductase | c8paqB_ | POXA 3b laccase small subunit | 71.6% | 75% | |  |
| Gene\|44023 | transport protein | c2hzqA_ | Apolipoprotein D | 99.1% | 79% | |  |
| Gene\|44019 | transport protein | c2hzqA_ | Apolipoprotein D | 99.1% | 79% | |  |
| Gene\|91264 | dna binding protein | c7qenF_ | Condensin complex subunit 1 | 62.8% | 46% | |  |
| Gene\|15775 | hydrolase | c3pjaL_ | translin-associated protein X | 10.7% | 15% | |  |
| Gene\|9657 | ligase | c4jghD_ | Cullin-5 | 54.9% | 48% | |  |
| Gene\|119663 | signaling protein | c5yylA_ | Major royal jelly protein 1 | 100.0% | **8787%** | |  |
| Gene\| 72613 | virus | c7bzuE_ | KRM 1 | 98.5% | 32% | |  |
| Gene\|23440 | signaling protein | c4xbmB_ | Delta-like protein 1 | 97.1% | 32% | |  |
| Gene\|17025 | chemotaxis | c2ch7A_ | Methyl-accepting chemotaxis protein | 98.0% | 43% | |  |
| Gene\|68132 | membrane protein | c7kiyC_ | High molecular weight rhoptry protein 3 | 15.8% | 18% | |  |
| Gene\|8967 | hydrolase/hydrolase  inhibitor | c4gi3B_ | Greglin | 94.9% | 48% | |  |
| Gene\|18854 | structural protein | c3fblA_ | Putative Uncharacterized protein | 64.2% | 41% | |  |
| Gene\|39089 | signaling protein | c3ja6H_ | Methyl-accepting chemotaxis protein 2 | 99.7% | 60% | |  |
| Gene\|105688 | hydrolase | c2oxeA_ | Pancreatic lipase-related protein 2 | 100% | 90% | |  |
| Gene\|44043 | hydrolase | c3vimA_ | Beta-glucosidase | 100% | 95% | |  |
| Gene\|49370 | isomerase | c2q2eB_ | Type 2 DNA topoisomerase 6 subunit B | 12.5% | 13% | |  |
| Gene\|104495 | isomerase | c4orwA_ | Prostaglandin-H2 D-isomerase | 99.7% | 72% | |  |
| Gene\|123673 | lyase | c3ea3A_ | 1-phosphatidylinositol phosphodiesterase | 100% | 75% | |  |
| Gene\|122466 | hydrolase/hydrolase  inhibitor | c4gi3B_ | Greglin | 66.2% | 7% | |  |
| Gene\|63545 | protein fibril | c6i2oA_ | Type IV pilin PilE1 | 13.5% | 31% | |  |
| Gene\|19684 | motor protein | c3rjsA_ | Dynein light chain motor protein | 73% | 52% | |  |
| Gene\|49972 | cytosolic protein | c7ztcA_ | actin, cytoplasmic 1 | 100% | 100% | |  |
| Gene\|5935 | cell invasion | c2p7nA_ | Pathogenicity island 1 effector protein | 52% | 49% | |  |
| Gene\|37280 | membrane protein | c8em4A_ | Low-density lipoprotein receptor | 96% | 53% | |  |
| Gene\|17926 | hydrolase inhibitor | c3pzfA_ | Serpin 2 | 100% | 58% | |  |
| Gene\|9418 | transport protein | c2hzrA_ | Apolipoprotein D | 99.8% | 74% | |  |
| Gene\|43644 | hydrolase | c7kdvB_ | Lysossomal protective protein | 100% | 83% | |  |
| Gene\|96 | hydrolase | c7mp4A_ | Carboxylesterase-24 | 100% | 89% | |  |
| Gene\|123335 | virus | c6snwE_ | Kremen protein 1 | 84% | 49% | |  |
| Gene\|14570 | membrane protein | c8em4A_ | Low-density lipoprotein | 97.2% | 66% | |  |
| Gene\|86762 | blood clotting | c7tppA_ | Prothrombin | 100% | 79% | |  |
| Gene\|75477 | hydrolase/hydrolase inhibitor | c4gi3B_ | Greglin | 85.2% | 21% | |  |
| Gene\|25728 | signaling protein | c6ml1D_ | Ubiquitin variant 15.1a | 92.6% | 73% | |  |
| Gene\|98956 | biosynthetic protein | c6yrzA_ | Fatty acid photodecarboxylase, chloroplastic | 100% | 84% | |  |
| Gene\|939 | lyase | c4rv3A_ | 1-phosphatidylinositol phosphodiesterase | 100% | 98% | |  |
| Gene\|30185 | lipocalin | c1gkaB_ | Crustacyanin A2 subunit | 99.8% | 82% | |  |
| Gene\|63746 | lipid binding protein | c1z24A_ | Insecticyanin A form | 93.1% | 88% | |  |
| Gene\|28211 | fluorescent protein | c7vnsA_ | Sandercyanin Fluorescent protein | 99.1% | 69% | |  |
| Gene\|34607 | dna binding  protein/dna | c7y8rB_ | Histone H4 | 98.6% | 91% | |  |
| Gene\|87793 | signaling protein | c5fwsA_ | Kremen protein 1 | 97.9% | 29% | |  |
| Gene\|90054 | signaling protein | c9b3nA_ | Neurogenic locus notch homolog protein 1 | 96% | 25% | |  |
| Gene\|9962 | structural protein | c8iviW_ | Tubulin alpha-3 chain | 100% | 98% | |  |
| Gene\|125314 | structural protein | c8iyjW_ | Tubulin alpha-3 chain | 100% | 98% | |  |
| Gene\|24312 | hydrolase | c1fcqA_ | Hyaluronoglucosaminidase | 100% | 87% | |  |
| Gene\|50887 | hydrolase/hydrolase inhibitor | c4bd9B_ | Carboxypeptidase inhibitor SMCI | 99.8% | 84% | |  |
| Gene\|57421 | amine-binding protein | c4ge1A_ | Biogenic amine-binding protein | 99.9% | 80% | |  |
| Gene\|50914 | isomerase | c3hjbD_ | Glucose-6-phosphate isomerase | 45.6% | 10% | |  |
| Gene\|125317 | structural protein | c8iyjW_ | Tubulin alpha-3 chain | 100% | 100% | |  |
| Gene\|99484 | dna binding protein/dna | c8x1cI_ | Helicase SRCAP | 45.1% | 26% | |  |
| Gene\|29301 | endocytosis/ exocytosis | c1hs7A_ | Syntaxin VAM3 | 41.9% | 14% | |  |
| Gene\|26346 | gene regulation | c8pp6E_ | Histone H3 (Fragment) | 99.8% | 67% | |  |
| Gene\|96176 | cell cycle | c6qj2B_ | Condensin complex subunit 2 | 28% | 34% | |  |
| Gene\|104521 | translation | c6ra9B_ | Elongation factor 1-alpha 2 | 100% | 98% | |  |
| Gene\|34859 | transport protein | c2hzqA_ | Apolipoprotein D | 99.9% | 81% | |  |
| Gene\|93867 | structural protein | c5xm04_ | Histone H3.3 | 99.7% | 71% | |  |
| Gene\|25376 | chaperone | c6nr8B_ | Prefoldin subunit 2 | 19.1% | 21% | |  |
| Gene\|67281 | ligand binding protein | c1kxoA_ | DigA16 | 98% | 74% | |  |
| Gene\|53847 | transferase | c6x5hA_ | Alpha-(1,6)-fucosyltransferase | 99.4% | 73% | |  |
| Gene\|89557 | protein transport | c3nf5A_ | Nucleoporin NUP116 | 26.1% | 61% | |  |
| Gene\|48825 | membrane protein | c6j5iA_ | ATP synthase subunit alpha, mitochondrial | 100% | 89% | |  |
| Gene\|35274 | membrane protein | c8em4A_ | Low-density lipoprotein receptor-related protein 2 | 97.7% | 82% | |  |
| Gene\|68184 | dna binding protein | c8rhnG_ | cDNA FLJ55172 | 61.6% | 49% | |  |
| Gene\|115476 | blood clotting | c8eo2A_ | Lufaxin | 72.6% | 35% | |  |
| Gene\|123671 | hydrolase | c1aodA_ | Phosphatidylinositol-specific phospholipase C | 99.7% | 92% | |  |
| Gene\|1053 | signaling protein | c5fwsA_ | Kremen protein 1 | 97.6% | 47% | |  |
| Gene\|49970 | protein fibril | c6iugA_ | pollen F-actin | 99.8% | 100%% | |  |
| Gene\|94739 | cytokine/de novo protein | c5knhB_ | IL13 | 25.1% | 29% | |  |
| Gene\|122583 | hydrolase | c4ud9A_ | Thrombin heavy chain | 100% | 74% | |  |
| Gene\|63749 | lipid binding protein | c1z24A_ | Insecticyanin A form | 98.2% | 76% | |  |
| Gene\|124737 | virus | c7bzuE_ | KRM1 | 99% | 38% | |  |
| Gene\|68129 | protein binding, dna binding protein | c2kn8A_ | DNA clevage and packaging protein large subunit,UL89 | 6.3% | 16% | |  |
| gene\|33249 | viral protein/immune system | [c7u0lA_](https://www.sbg.bio.ic.ac.uk/phyre2/phyre2_output/b158f4e823684c6f/summary.html#c7u0lA_) | Aminopeptidase N | 100 % | 90 % | |  |
| Gene_64061 | ribosome | [c7mqaV_](https://www.sbg.bio.ic.ac.uk/phyre2/phyre2_output/2cd9c8844b7846d5/summary.html#c7mqaV_) | U3 small nucleolar RNA-associated protein 4 homolog | 100 % | 95 % | |  |
| Gene_8242 | hormone | c8i2gE_ | Follicle-stimulating | 100 % | 50 % | |  |
| Gene_15229 | cell adhesion | c3ubhA_ | Neural-cadherin | 99.7 % | 14 % | |  |
| Gene_34230 | hydrolase | [c3vimA_](https://www.sbg.bio.ic.ac.uk/phyre2/phyre2_output/a69f10178cd41507/summary.html#c3vimA_) | Beta-glucosidase | 100 % | 98 % | |  |
| Gene_99411 | contractile protein | [c1sjjB_](https://www.sbg.bio.ic.ac.uk/phyre2/phyre2_output/f6ed71629095a012/summary.html#c1sjjB_) | actinin | 99.9 % | 13 % | |  |
| Gene_127197 | hydrolase | [c4madB_](https://www.sbg.bio.ic.ac.uk/phyre2/phyre2_output/096f420b7f3e3edf/summary.html#c4madB_) | Beta-galactosidase | 100 % | 83 % | |  |
| Gene_86414 | nuclear protein/dna | [c7y7iA_](https://www.sbg.bio.ic.ac.uk/phyre2/phyre2_output/d3102fc2a1abfc01/summary.html#c7y7iA_) | Histone H3.1, Histone H3-like centromeric protein A | 99.8 % | 48 % | |  |
| Gene_2383 | hydrolase/hydrolase inhibitor | [c3fp8A_](https://www.sbg.bio.ic.ac.uk/phyre2/phyre2_output/88d5b1f6cf5352ec/summary.html#c3fp8A_) | Anionic trypsin-2 | 100 % | 71 % | |  |
| Gene_115361 | membrane protein | [c7p2yB_](https://www.sbg.bio.ic.ac.uk/phyre2/phyre2_output/60ec206524a14420/summary.html#c7p2yB_) | ATP synthase subunit | 100 % | 97 % | |  |
| Gene_22707 | oxidoreductase/oxidoreductase inhibitor | [c3nksA_](https://www.sbg.bio.ic.ac.uk/phyre2/phyre2_output/ebf442bc676af3ef/summary.html#c3nksA_) | Protoporphyrinogen oxidase | 100 % | 97 % | |  |
| Gene_47299 | RNA binding protein | [c5ubaA_](https://www.sbg.bio.ic.ac.uk/phyre2/phyre2_output/a3a937940d94a7ef/summary.html#c5ubaA_) | RNA pseudouridylate synthase domain-containing protein 4 | 100 % | 72 % | |  |
| Gene_24673 | oxidoreductase/oxidoreductase inhibitor | [c7sv2A_](https://www.sbg.bio.ic.ac.uk/phyre2/phyre2_output/eff217c652940a64/summary.html#c7sv2A_) | Cytochrome P450 3A5 | 100 % | 86 % | |  |
| Gene_96371 | blood clotting | c1ei3B_ | FIBRINOGEN | 47.4 % | 7 % | |  |
| Gene_29652 | ribosome | [c7aspP_](https://www.sbg.bio.ic.ac.uk/phyre2/phyre2_output/e2fc0278ca9fa67b/summary.html#c7aspP_) | 30S ribosomal protein S15 | 100 % | 100 % | |  |
| Gene_20855 | transferase | [c2g7mA_](https://www.sbg.bio.ic.ac.uk/phyre2/phyre2_output/10cf4c236b0e11a0/summary.html#c2g7mA_) | putative ornithine carbamoyltransferase | 99.6 % | 98 % | |  |
| Gene_14682 | oxidoreductase | [c6c93A_](https://www.sbg.bio.ic.ac.uk/phyre2/phyre2_output/6af6f52adaadd2f4/summary.html#c6c93A_) | Cytochrome P450 4B1 | 100 % | 93 % | |  |
| Gene_71935 | ransferase, hydrolase/rna/dna | [c4ol8B_](https://www.sbg.bio.ic.ac.uk/phyre2/phyre2_output/9a4d8826200c7039/summary.html#c4ol8B_) | Reverse transcriptase/ribonuclease H | 100 % | 30 % | |  |
| Gene_39794 | hydrolase | [c3zfdA_](https://www.sbg.bio.ic.ac.uk/phyre2/phyre2_output/1b5f7bb05f74b48c/summary.html#c3zfdA_) | CHROMOSOME-ASSOCIATED KINESIN KIF4 | 100 % | 21 % | |  |
| Gene_44006 | transport protein | [c4c0pD_](https://www.sbg.bio.ic.ac.uk/phyre2/phyre2_output/c6ab6805476ba985/summary.html#c4c0pD_) | TRANSPORTIN-3 | 92.8 % | 55 % | |  |
| Gene_124425 | protein transport | [c2a2fA_](https://www.sbg.bio.ic.ac.uk/phyre2/phyre2_output/172e3469c61cd14d/summary.html#c2a2fA_) | Exocyst complex component Sec15 | 100 % | 38 % | |  |
| Gene_95308 | transport protein | [c3w68B_](https://www.sbg.bio.ic.ac.uk/phyre2/phyre2_output/6525e02781b15aaf/summary.html#c3w68B_) | Alpha-tocopherol transfer protein | 100 % | 79 % | |  |
| Gene_118859 | protein transport | [c6jm5A_](https://www.sbg.bio.ic.ac.uk/phyre2/phyre2_output/a86ebc46634172d3/summary.html#c6jm5A_) | TBC1 domain family member 23 | 7.7 % | 20 % | |  |
| Gene_116937 | replication, transferase/dna | [c2pzsG_](https://www.sbg.bio.ic.ac.uk/phyre2/phyre2_output/89872952f7afae8a/summary.html#c2pzsG_) | DNA polymerase | 100 % | 37 % | |  |
| Gene_56104 | dna binding protein | [c7zygA_](https://www.sbg.bio.ic.ac.uk/phyre2/phyre2_output/9d6bba9ff510e53f/summary.html#c7zygA_) | X-ray repair cross-complementing protein 6 | 100 % | 82 % | |  |
| Gene_82442 | transferase | [c1h3dA_](https://www.sbg.bio.ic.ac.uk/phyre2/phyre2_output/e48d3abf45f03f5d/summary.html#c1h3dA_) | ATP-PHOSPHORIBOSYLTRANSFERASE | 4.7 % | 3 % | |  |
| Gene_74414 | ligase | [c3ig5A_](https://www.sbg.bio.ic.ac.uk/phyre2/phyre2_output/ed851e36d722071f/summary.html#c3ig5A_) | Glutamate-cysteine ligase | 100 % | 94 % | |  |
| Gene_40922 | motor protein | [c7kogA_](https://www.sbg.bio.ic.ac.uk/phyre2/phyre2_output/3353f1ff722fec30/summary.html#c7kogA_) | Myosin heavy chain isoform Mhc_X1 | 96.7 % | 29 % | |  |
| Gene_30710 | oxidoreductase | [c6c93A_](https://www.sbg.bio.ic.ac.uk/phyre2/phyre2_output/94cf6bbed4cbd9e6/summary.html#c6c93A_) | Cytochrome P450 4B1 | 99.9 % | 86 % | |  |
| Gene_113908 | signaling protein | c3wpqA_ | Rac GTPase-activating protein 1 | 99.9 % | 33 % | |  |
| Gene_32169 | rna binding protein | c1zu1A_ | RNA binding protein ZFa | 95 % | 6 % | |  |
| Gene_41192 | membrane protein | c8h9lF_ | ATP synthase subunit beta, mitochondrial | 100 % | 94 % | |  |
| Gene_41187 | hydrolase | c5lqyH_ | ATP synthase beta subunit | 100 % | 92 % | |  |
| Gene_60771 | dna binding protein | [c8deiB_](https://www.sbg.bio.ic.ac.uk/phyre2/phyre2_output/9970dfe8cfb06734/summary.html#c8deiB_) | Maltodextrin-binding protein, Chromatin assembly factor 1 subunit p90 fusion | 90.2 % | 16 % | |  |
| Gene_30988 | oxidoreductase | [c6c93A_](https://www.sbg.bio.ic.ac.uk/phyre2/phyre2_output/6a9826dc75f7c083/summary.html#c6c93A_) | Cytochrome P450 4B1 | 99.8 % | 96 % | |  |
| Gene_39434 | signaling protein | [c5yylA_](https://www.sbg.bio.ic.ac.uk/phyre2/phyre2_output/04bf6d6ffd3e9e47/summary.html#c5yylA_) | Major royal jelly protein 1 | 100 % | 90 % | |  |
| Gene_50713 | membrane protein | [c6c5wA_](https://www.sbg.bio.ic.ac.uk/phyre2/phyre2_output/ab5c9453f8676726/summary.html#c6c5wA_) | calcium uniporter | 12.7 % | 50 % | |  |
| Gene_69580 | hormone/growth factor | [c1rj8A_](https://www.sbg.bio.ic.ac.uk/phyre2/phyre2_output/c4fbc945581fe453/summary.html#c1rj8A_) | ectodysplasin-A isoform EDA-A2 | 99.9 % | 35 % | |  |
| Gene_70051 | hydrolase | [c1qidA_](https://www.sbg.bio.ic.ac.uk/phyre2/phyre2_output/7c7b9ecbdbbe33fe/summary.html#c1qidA_) | ACETYLCHOLINESTERASE | 100 % | 98 % | |  |
| Gene_28090 | atp synthase | [c1qo1D_](https://www.sbg.bio.ic.ac.uk/phyre2/phyre2_output/854792b66502c690/summary.html#c1qo1D_) | ATP SYNTHASE BETA CHAIN | 100 % | 86 % | |  |
| Gene_117495 | transcription | [c8b3dS_](https://www.sbg.bio.ic.ac.uk/phyre2/phyre2_output/80c86feef150e592/summary.html#c8b3dS_) | UV-stimulated scaffold protein A | 100 % | 42 % | |  |
| Gene_109751 | isomerase/dna | [c5eixA_](https://www.sbg.bio.ic.ac.uk/phyre2/phyre2_output/4d822d9637a7d9cf/summary.html#c5eixA_) | DNA topoisomerase 4 subunit B, DNA topoisomerase 4 subunit A | 99.7 % | 66 % | |  |
| Gene_122335 | hydrolase/rna/dna | [c4z7kB_](https://www.sbg.bio.ic.ac.uk/phyre2/phyre2_output/1ef204d73d7e97fb/summary.html#c4z7kB_) | Cas6b | 66.6 % | 38 % | |  |
| Gene_109233 | nuclear protein | [c6dztD_](https://www.sbg.bio.ic.ac.uk/phyre2/phyre2_output/9cb6b8bc5c01ef2c/summary.html#c6dztD_) | Histone H2B | 99.3 % | 76 % | |  |
| Gene_97354 | transferase | [c2g7mA_](https://www.sbg.bio.ic.ac.uk/phyre2/phyre2_output/a3c55543c033cca8/summary.html#c2g7mA_) | putative ornithine carbamoyltransferase | 100 % | 97 % | |  |
| Gene_111572 | structural protein/dna | [c3waaC_](https://www.sbg.bio.ic.ac.uk/phyre2/phyre2_output/5cab32ddbffb1dca/summary.html#c3waaC_) | Histone H2A.V | 99.2 % | 82 % | |  |
| Gene_97045 | protein transport | [c3zlcA_](https://www.sbg.bio.ic.ac.uk/phyre2/phyre2_output/9dad6aba000d3478/summary.html#c3zlcA_) | ER-DERIVED VESICLES PROTEIN ERV41 | 100 % | 54 % | |  |
| Gene_75398 | virus | [c6snwE_](https://www.sbg.bio.ic.ac.uk/phyre2/phyre2_output/8fb9e8b6adc667ff/summary.html#c6snwE_) | Kremen protein 1 | 99 % | 59 % | |  |
| Gene_101940 | lyase | [c4rv3A_](https://www.sbg.bio.ic.ac.uk/phyre2/phyre2_output/7869044a098d4834/summary.html#c4rv3A_) | 1-phosphatidylinositol phosphodiesterase | 99.9 % | 91 % | |  |
| Gene_13620 | virus | [c6snwE_](https://www.sbg.bio.ic.ac.uk/phyre2/phyre2_output/eeeb3c58c4157e34/summary.html#c6snwE_) | Kremen protein 1 | 97.7 % | 78 % | |  |
| Gene_105675 | ribosome | [c8b6zA_](https://www.sbg.bio.ic.ac.uk/phyre2/phyre2_output/1e820c488f7dabf7/summary.html#c8b6zA_) | Elongation factor 1-alpha 2 | 100 % | 98 % | |  |
| Gene_59599 | virus | [c7bzuE_](https://www.sbg.bio.ic.ac.uk/phyre2/phyre2_output/fbcbb48381a7f9fd/summary.html#c7bzuE_) | KRM1 | 98.9 % | 75 % | |  |
| Gene_69793 | hydrolase | [c8ssiA_](https://www.sbg.bio.ic.ac.uk/phyre2/phyre2_output/076034337f8b9a1a/summary.html#c8ssiA_) | Deubiquitinase TssM | 84.8 % | 76 % | |  |
| Gene_16116 | virus | [c7bzuE_](https://www.sbg.bio.ic.ac.uk/phyre2/phyre2_output/5b9bdc6db3752d33/summary.html#c7bzuE_) | KRM1 | 97.1 % | 16 % | |  |
| Gene_8808 | translation | [c6ra9B_](https://www.sbg.bio.ic.ac.uk/phyre2/phyre2_output/d8a9ebfbf5bbac36/summary.html#c6ra9B_) | Elongation factor 1-alpha 2 | 100 % | 98 % | |  |
| Gene_54186 | metal binding protein | [c7wlfA_](https://www.sbg.bio.ic.ac.uk/phyre2/phyre2_output/f1f37cf88590320d/summary.html#c7wlfA_) | Serum albumin | 99.9 % | 80 % | |  |
| Gene_30648 | signaling protein | [c6uktE_](https://www.sbg.bio.ic.ac.uk/phyre2/phyre2_output/a4ab16b155d7cbc2/summary.html#c6uktE_) | NB8119 | 9.1 % | 27 % | |  |
| Gene_44311 | lyase | [c3nwrA_](https://www.sbg.bio.ic.ac.uk/phyre2/phyre2_output/546e1e52aaacb870/summary.html#c3nwrA_) | A rubisco-like protein | 12.8 % | 53 % | |  |
| Gene_47672 | hydrolase/hydrolase inhibitor | [c4gi3B_](https://www.sbg.bio.ic.ac.uk/phyre2/phyre2_output/4a28ea4ad1e4b377/summary.html#c4gi3B_) | Greglin | 95. 5 % | 19 % | |  |
| Gene_29116 | gene regulation | [c8pp6G_](https://www.sbg.bio.ic.ac.uk/phyre2/phyre2_output/21b2a52a1231b6f2/summary.html#c8pp6G_) | Histone H2A | 98.9 % | 75 % | |  |
| Gene_59666 | structural protein | [c5icaC_](https://www.sbg.bio.ic.ac.uk/phyre2/phyre2_output/e1becec7ec991026/summary.html#c5icaC_) | Putative U3 snoRNP protein | 61.5 % | 31 % | |  |
| Gene_123331 | signaling protein | [c5mvxA_](https://www.sbg.bio.ic.ac.uk/phyre2/phyre2_output/68e26b9e825edc73/summary.html#c5mvxA_) | Delta-like protein 4 | 98.3 % | 80 % | |  |
| Gene_11439 | blood clotting | [c8eo2A_](https://www.sbg.bio.ic.ac.uk/phyre2/phyre2_output/2e25f0f2d91449e8/summary.html#c8eo2A_) | Lufaxin | 80.8 % | 69 % | |  |
| Gene_2656 | oxidoreductase | [c4gieA_](https://www.sbg.bio.ic.ac.uk/phyre2/phyre2_output/a9b0cc6d6f9d13dd/summary.html#c4gieA_) | Prostaglandin F synthase | 27 % | 21 % | |  |
